# Interplay between mitochondria and diet mediates pathogen and stress resistance in *Caenorhabditis elegans*

**DOI:** 10.1371/journal.pgen.1008011

**Published:** 2019-03-13

**Authors:** Alexey V. Revtovich, Ryan Lee, Natalia V. Kirienko

**Affiliations:** Department of BioSciences, Rice University, Houston TX, United States of America; The University of Texas Health Science Center at Houston, UNITED STATES

## Abstract

Diet is a crucial determinant of organismal biology; interactions between the host, its diet, and its microbiota are critical to determining the health of an organism. A variety of genetic and biochemical means were used to assay stress sensitivity in *C*. *elegans* reared on two standard laboratory diets: *E*. *coli* OP50, the most commonly used food for *C*. *elegans*, or *E*. *coli* HT115, which is typically used for RNAi-mediated gene knockdown. We demonstrated that the relatively subtle shift to a diet of *E*. *coli* HT115 had a dramatic impact on *C*. *elegans*’s survival after exposure to pathogenic or abiotic stresses. Interestingly, this was independent of canonical host defense pathways. Instead the change arises from improvements in mitochondrial health, likely due to alleviation of a vitamin B12 deficiency exhibited by worms reared on an *E*. *coli* OP50 diet. Increasing B12 availability, by feeding on *E*. *coli* HT115, supplementing *E*. *coli* OP50 with exogenous vitamin B12, or overexpression of the B12 transporter, improved mitochondrial homeostasis and increased resistance. Loss of the methylmalonyl-CoA mutase gene *mmcm-1*/*MUT*, which requires vitamin B12 as a cofactor, abolished these improvements, establishing a genetic basis for the *E*. *coli* OP50-incurred sensitivity. Our study forges a mechanistic link between a dietary deficiency (nutrition/microbiota) and a physiological consequence (host sensitivity), using the host-microbiota-diet framework.

## Author summary

Vitamin B12 deficiency affects ~10–40% of US adults, causing a range of health issues ranging from anemia to neurological defects. Here we provide a mechanistic link between dietary B12 deficiency and mitochondrial dysfunction. Our data indicate that B12 supports clearance of propionate, an intermediate of branched chain amino acid metabolism. Excess propionate compromises mitochondrial homeostasis, increasing susceptibility to abiotic stresses and bacterial pathogenesis. Importantly, this deficiency is absent when worms are fed a diet of *E*. *coli* HT115. *C*. *elegans* reared on *E*. *coli* OP50 are predisposed to a variety of health defects, including increased sensitivity to bacterial pathogens and stresses; these subtle phenotypes may be difficult to recapitulate under conventional RNAi feeding conditions with HT115, introducing unexpected experimental confounds. These findings reinforce the importance of considering the host-microbiota-nutrient axis when using this model organism.

## Introduction

Like it's genome, an organism's diet and microbiota tremendously influence its life history. Attempts to understand this have led to the development of the host-microbiota-nutrition axis model [1]. Although an avalanche of literature has reported on variations in the microbiota, diet, and genomes of model organisms, mechanistic understanding has lagged considerably behind description. The dynamic and complex interactions between these three systems form something of a "biological three-body problem". For example, host genetics and microbiotic metabolism influence the nutritional value of the diet consumed [2,3]. Simultaneously, the host's environment determines its initial susceptibility to microbial colonization [4]. Despite the difficulty, it is crucial to establish a mechanistic understanding of these interrelationships to properly understand host healthspan under normal and stress conditions.

*Caenorhabditis elegans* offers a tantalizing system for simplifying these studies without sacrificing the ability to make discoveries useful for more complex organisms. Generation of gnotobiotic worms is simple, efficient, and inexpensive, only requiring treatment of gravid adults with hypochlorite to release eggs that will hatch into microbe-free larvae. *C*. *elegans* can then simply be transferred to agar plates seeded with a wide-variety of bacteria (either a single strain or a customized mixture). Generally, the host readily consumes the bacteria provided. In its most reductionist form, the host-microbiota-nutrition axis of *C*. *elegans* can be collapsed to a binary system comprised of only two species, both of which are genetically tractable: *C*. *elegans*, the bacterivorous host, and *E*. *coli*, which serves as food. Although *C*. *elegans* is typically not colonized by *E*. *coli* the way that humans are (at least not early in its life), Cabreiro and Gems make a compelling argument that *E*. *coli* serves at least most of the functions for *C*. *elegans* that a conventional microbiota confers to its host, including protection against pathogens, immune maturation, digestive aid, vitamin production, and xenobiotic metabolism [5]. So by at least some definitions, *E*. *coli* can comprise a microbiota for *C*. *elegans* as well.

Even this simple binary system has yielded a number of significant insights into the complicated interactions between host, diet, and microbiota. Different diets profoundly affect the worm transcriptome [6], metabolome [7], intestinal fat storage [8] and lifespan [6–9]. The metabolic activity of the bacterial food also influences the metabolism of *C*. *elegans* [10,11]. For example, *C*. *elegans* feeding on *Bacillus subtilis* were observed to have a greater lifespan than their *E*. *coli*-fed counterparts [12]. This effect was shown to be a consequence of the host utilizing bacterially-derived nitric oxide to stimulate expression of HSF-1 and DAF-16, which increase lifespan [13]. In contrast, *C*. *elegans*'s lifespan can be shortened by bacterial folate metabolism [14,15], probably via increased *S*-adenosylmethionine (SAM) synthesis [16]. The link between SAM and lifespan was demonstrated by an elegant study that showed that metformin, by limiting SAM production and stimulating AMPK activation, increases *C*. *elegans* lifespan [16]. Unfortunately, this is unlikely to be a viable method to increase healthspan; SAM is a crucial methyl group donor necessary for a wide variety of cellular activities. For example, *C*. *elegans sams-1 (RNAi)* mutants exhibit constitutive immune activation, but despite this, they show increased sensitivity to *Pseudomonas aeruginosa* [17].

Like humans, *C*. *elegans* is incapable of creating several essential vitamins, including vitamin B12, and they must be obtained from their diet. Vitamin B12 exists as two biologically active, readily interconvertible vitamers, methylcobalamin and adenosylcobalamin. These compounds are involved in the biosynthesis of methionine and the conversion of methylmalonyl-CoA to succinyl-CoA, respectively. This latter pathway is required for the proper breakdown of propionate and branched-chain amino acids, the failure of which is associated with mitochondrial dysfunction [18,19]. Serious consequences have been associated with severe vitamin B12 deficiency in *C*. *elegans*, including infertility, slowed growth, and shortened lifespan [20,21]. In both *C*. *elegans* and mammals, B12 deficiency causes toxic intermediates to accumulate [22,23]. Interestingly, the most common *C*. *elegans* lab diet, *E*. *coli* OP50, results in a mild, chronic vitamin B12 deficiency [6,21].

Through a variety of assays, we discovered that the vitamin B12 deficiency caused by a diet of *E*. *coli* strain OP50 disrupts mitochondrial homeostasis, sensitizing the host to infection and a variety of abiotic stresses. B12 supplementation, even in the absence of living bacteria, increased resistance without affecting lifespan. Genetic analysis mapped this phenotype to the methylmalonyl/succinyl-CoA breakdown pathway, where vitamin B12 serves as a cofactor for MMCM-1/MUT. Our findings provide a mechanistic link between diet, cellular homeostasis, and organismal health.

## Results

### A diet of *E*. *coli* strain HT115 confers resistance to multiple bacterial pathogens

**Fig 1:**
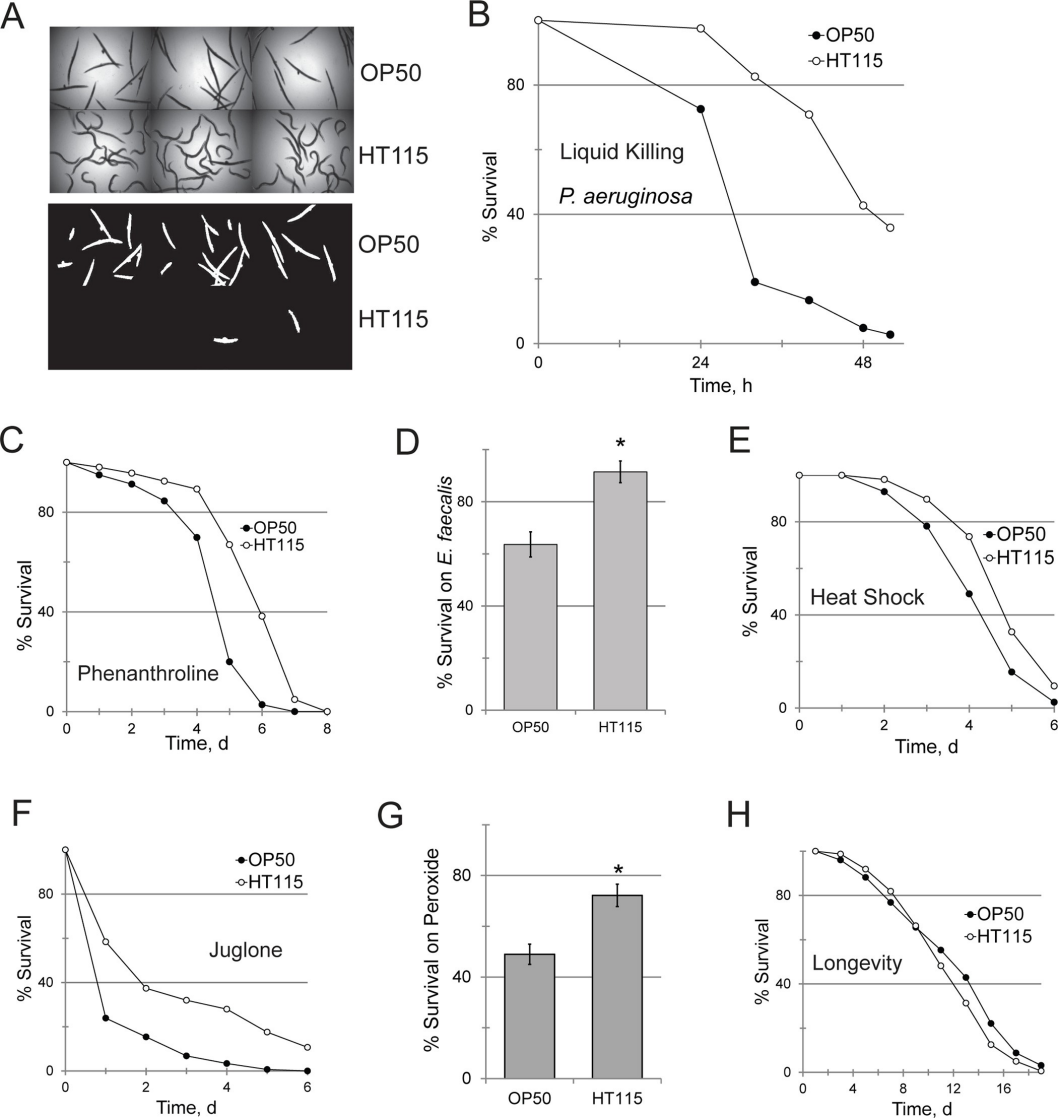
*E coli* HT115 increases *C*. *elegans'* resistance to stresses. **(A)** Representative bright-field (top) and fluorescent (bottom) images of *glp-4(bn2) C*. *elegans* exposed to *P*. *aeruginosa* PA14 after feeding on *E*. *coli* OP50 or *E*. *coli* HT115. Fraction of surviving worms (here and elsewhere) was inferred based on staining with Sytox Orange, a cell impermeant dye. **(B)** Time course of Liquid Killing of *C*. *elegans* as in **(A)**. **(C-G)** Survival of OP50- and HT115-fed *glp-4(bn2)* worms after acute phenanthroline poisoning (100 μM) **(C)**, infection with *E*. *faecalis* OG1RF **(D)**, or exposure to hyperthermic (30°C) **(E)** or oxidative stresses from juglone (120 μM) **(F)** or hydrogen peroxide (2 mM) **(G)**. **(H)** Lifespan of *C*. *elegans* fed with *E*. *coli* OP50 or *E*. *coli* HT115. *p*-value for **(B-G)** <0.01, **(H)** > 0.05 (N.S.). *p-*values were calculated using log-rank test **(B**,**C,E,F,H)** or Student’s *t*-test **(D,G)**. *—*p*<0.01. At least 10 wells, each containing 20 worms, were used per replicate for **(A,B,D,G)**. At least 50 worms per plate, three plates per condition per replicate were used for **(C,E,F,H)**. At least three biological replicates were performed for each experiment.

While characterizing a *C*. *elegans-P*. *aeruginosa* Liquid Killing assay [24,25], we made the unexpected observation that diet plays a large role in the hosts' survival after exposure to *P*. *aeruginosa* strain PA14. For example, median survival time of *glp-4(bn2)* worms reared on *E*. *coli* HT115 was 48h, while worms reared on *E*. *coli* OP50 showed median survival closer to 30h (**Fig 1A and 1B**). *E*. *coli* HT115 is used ubiquitously for RNAi in *C*. *elegans*; this strain is the host for the two largest, publicly available whole-genome RNAi libraries [26,27]. Importantly, the increase in resistance to *P*. *aeruginosa* PA14 occurred regardless of whether either *E*. *coli* strain carried the L4440 RNAi plasmid vector (**S1A Fig**). Pathogenesis in this assay depends on intoxication with the siderophore pyoverdine, which removes iron from the *C*. *elegans* host [28,29]. 1,10-phenanthroline, a small synthetic chelator, mimics many of the aspects of siderophore-mediated killing [29,30], so resistance of *E*. *coli* HT115-fed worms to this compound was also assayed. *glp-4*(*bn2*) worms fed HT115 were more resistant to phenanthroline than their OP50-fed counterparts (**Fig 1C**).

We have previously shown that exogenous iron strongly limited the ability of pyoverdine or 1,10-phenanthroline to cause host death [24]. Therefore, one possibility is that *E*. *coli* HT115 may be indirectly increasing resistance by providing more iron to *C*. *elegans*. To test this hypothesis, total host iron, using inductively-coupled plasma mass spectrometry, or iron (III), using a fluorometric method, were measured. In each assay, iron levels were indistinguishable, regardless of the food tested (**S1B and S1C Fig**).

Since these findings suggested that the deficiency was not related to iron, we suspected the sensitivity may also exist for other pathogens. To test this, *glp-4*(*bn2*) worms were reared on either *E*. *coli* OP50 or HT115, and then exposed to *Enterococcus faecalis* OG1RF, another human bacterial pathogen, in a recently-developed Liquid Killing assay [31]. To the best of our knowledge, pathogenesis in this assay is independent of siderophores. Interestingly, *E*. *coli* HT115 also increased survival of *C*. *elegans* infected with this pathogen by ~30% (**Fig 1D**). *E*. *faecalis* OG1RF is a gram positive pathogen that likely uses different pathogenic determinants than *P*. *aeruginosa* PA14 [32], suggesting that the mechanism of resistance has a broad spectrum of activity.

The ability of *E*. *coli* HT115 to confer resistance to *P*. *aeruginosa*-mediated slow killing was also tested. Unlike the liquid-based pathogenesis assays, no statistically significant difference was seen between worms reared on the two diets (**S2A Fig**). To ensure that the worms were not colonized differently, a strain of *P*. *aeruginosa* engineered to express DsRed was used to infect worms after they were reared on each diet. Again, no statistically significant difference was observed (**S2B and S2C Fig**). One possible explanation for this difference is that pathogenesis in this assay is thought to take place via intestinal colonization; the biological difference induced by a diet of *E*. *coli* HT115 may be irrelevant for this mechanism of pathogenesis.

### Diet-mediated sensitivity is independent of innate immunity

Substitution of *E*. *coli* OP50 with a variety of other bacterial foods (or dead *E*. *coli*) increases the lifespan of *C*. *elegans* [33–36]. This is often interpreted as evidence that *E*. *coli* OP50 is weakly pathogenic to *C*. *elegans* [37,38], opening the possibility that *E*. *coli* HT115 is less pathogenic than *E*. *coli* OP50 (at least under these conditions). In this case, it is likely that the immune response of *C*. *elegans* would differ between OP50 and HT115. Transcription of a number of *C*. *elegans* innate immune genes, regulated by PMK-1/p38, ZIP-2/bZIP, DAF-16/FOXO, FSHR-1/FSH, and SKN-1/Nrf [12,39–43] was surveyed. Synchronized *glp-4*(*bn2*) worms were reared on either *E*. *coli* OP50 or *E*. *coli* HT115 to the young adult stage and basal gene expression for these pathways was assessed. Gene expression levels were indistinguishable between food sources (**S3 Fig**).

We also assayed induction, rather than basal expression, of the innate immune pathways using RNAi. As noted above, RNAi in *C*. *elegans* is typically performed using *E*. *coli* HT115, in part because *E*. *coli* OP50 generally expresses dsRNA quite poorly [44]. Fortunately, an RNAseIII-deficient strain of OP50, called *xu363*, that efficiently produce dsRNA at levels comparable to *E*. *coli* HT115 has recently been engineered [45]. Comparisons of *cyc-1(RNAi)* in the two strains (*E*. *coli* OP50(*xu363*) and *E*. *coli* HT115) suggest that they exhibit comparable levels of gene knockdown (**S4 Fig**).

RNAi constructs for *pmk-1*, *zip-2*, *daf-16*, *fshr-1*, and *skn-1* were transferred from *E*. *coli* HT115 into *E*. *coli* OP50(*xu363*). *glp-4*(*bn2*) mutants were then reared on either *E*. *coli* OP50(*xu363*) or *E*. *coli* HT115 expressing each RNAi construct. At the young adult stage, pathogenesis in the Liquid Killing assay was tested. As expected, some gene disruptions altered the timing of death compared to bacteria containing an empty RNAi vector. For example, *daf-16* knockdown, which sensitizes worms to Liquid Killing [28], hastened death in each case. *daf-2(RNAi)*, which constitutively activates DAF-16/FOXO, prolonged survival. Importantly, worms fed *E*. *coli* HT115 survived longer than worms fed *E*. *coli* OP50(*xu363*), regardless of the gene targeted by RNAi (**S5 Fig**). Combined, these data indicate that the weak pathogenicity reported for *E*. *coli* OP50 is unlikely to be causing the increased sensitivity to *P*. *aeruginosa*, phenanthroline, and *E*. *faecalis*.

### *E*. *coli* HT115 also promotes resistance to abiotic stresses

Several innate immune pathways in *C*. *elegans* (such as those regulated by DAF-16/FOXO, SKN-1/Nrf, or the ESRE network) also promote survival during exposure to abiotic stresses, like heat or free-radical inducing chemicals. *glp-4*(*bn2*) worms were reared on *E*. *coli* OP50 or *E*. *coli* HT115 and then exposed as young adult worms to either heat shock, juglone, or hydrogen peroxide. In each case, *E*. *coli* HT115-fed worms survived better (**Fig 1E–1G**). Because increased stress or pathogen resistance is often associated with longer lifespan (which has led to the theory that the former is responsible for the latter), we compared the average lifespan of worms reared on these bacteria. However, *E*. *coli* HT115 did not increase host lifespan (**Fig 1H**).

### Increased resistance is not due to the *glp-4(bn2)* phenotype

Another possible explanation that emerged was the temperature-dependent sterility induced by the *glp-4(bn2)* phenotype. For technical reasons, it is necessary to induce sterility in worms for liquid-based killing assays [24]. The *glp-4(bn2)* mutation compromises a valyl aminoacyl tRNA synthetase (VARS-2) [46] which prevents development of the germline, but causes no other overt phenotype [47–49]. When the germline of *C*. *elegans* is removed, lifespan is extended in a DAF-16/FOXO-dependent fashion [50,51]. Since this transcription factor promotes broad-spectrum stress resistance, it was important to determine whether wild-type *C*. *elegans* also exhibited a difference in stress resistance when reared on *E*. *coli* HT115.

Wild-type N2 worms were reared on *E*. *coli* OP50 or *E*. *coli* HT115 and then subjected to Liquid Killing, propionate, or heat shock. Lifespans were also measured. In each case, results from wild-type worms recapitulated our findings from *glp-4(bn2)* mutants (**S6 Fig**).

### Transcriptome profiling implicates mitochondrial perturbations in OP50-induced stress sensitivity

**Fig 2:**
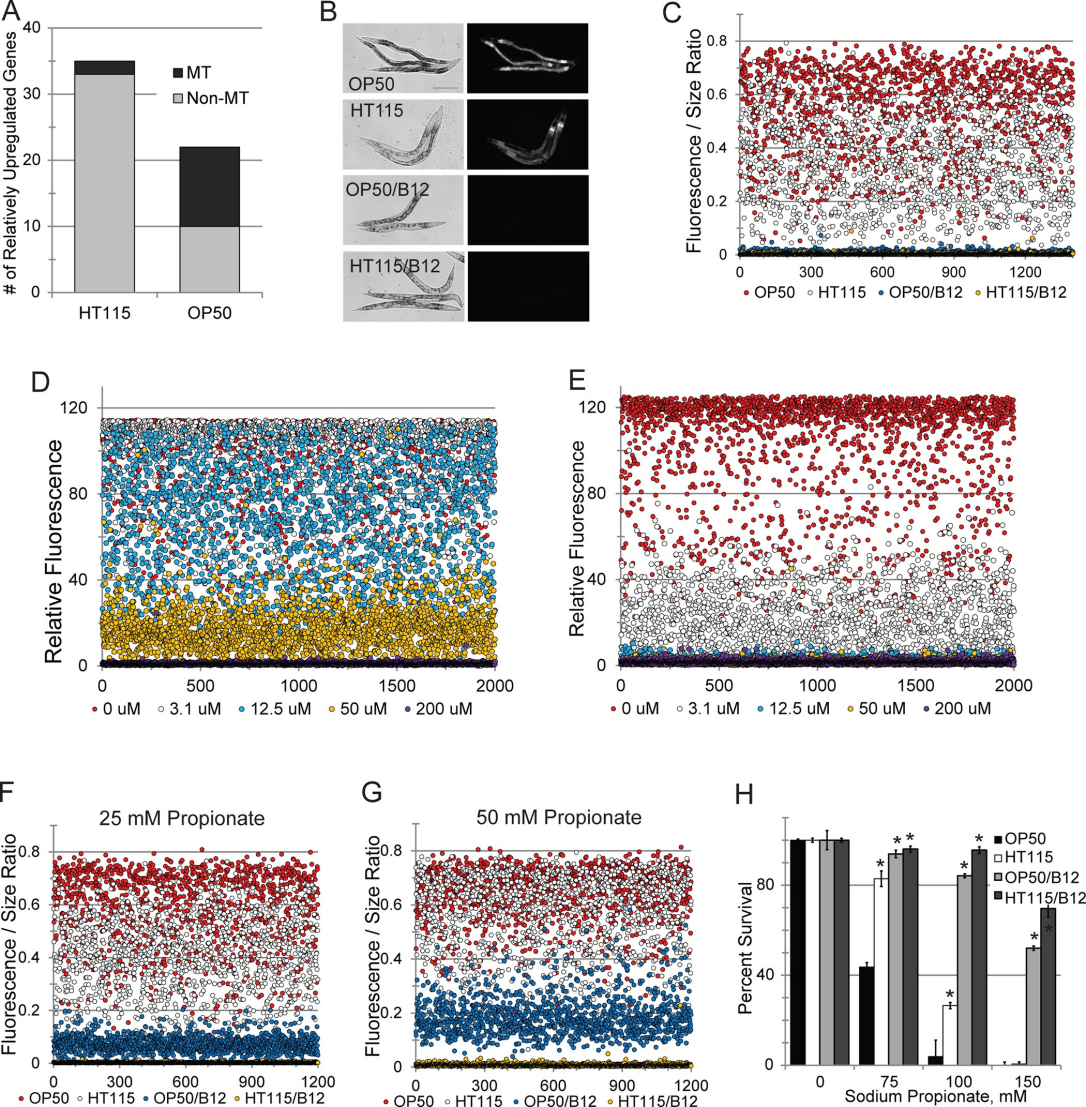
A diet of *E*. *coli* OP50 leads to vitamin B12 deficiency in *C*. *elegans*. **(A)** Number of genes upregulated in worms fed either *E*. *coli* HT115 or *E*. *coli* OP50 when normalized to the other food. Genes annotated as having a mitochondrial localization or function are shown in black, while non-mitochondrial genes are shown in grey. **(B)** Visualization of *acdh-1p*::GFP reporter fluorescence in worms fed *E*. *coli* OP50 or *E*. *coli* HT115, with or without methylcobalamin supplementation (200 ng/ml), as indicated (see **S7A Fig** for quantification). **(C)** *acdh-1p*::GFP expression in worms reared on *E*. *coli* OP50 or *E*. *coli* HT115 with or without methylcobalamin supplementation was measured using flow vermimetry. Fluorescence for each worm was normalized to its size (see **S7B Fig** for quantification). **(D,E)** *acdh-1p*::GFP fluorescence of worms reared on *E*. *coli* OP50 or *E*. *coli* HT115 supplemented with varying concentrations of methlycobalamin. **(F, G)** *acdh-1p*::GFP fluorescence of worms reared as in **(C)** with exogenous propionate. **(H)** Propionate toxicity in worms fed as in **(C)**. *—*p*<0.01, *p*-value was calculated based on Student’s *t*-test. For **(C-E)** at least 1500 worms were used for each replicate. For **(H)** approximately 70 worms were used per plate per condition per biological replicate. At least three biological replicates were performed for each experiment.

Without a clear explanation for the diet-induced difference in stress resistance, we turned toward microarray analysis to get an unbiased representation of transcriptional events. *glp-4(bn2)* worms were reared to young adult stage on either *E*. *coli* OP50 or *E*. *coli* HT115. RNA was collected and microarray analysis was performed essentially as previously described [29]. To our surprise, the number of genes differentially regulated was relatively small; only 35 genes showed upregulation between 2- and 8-fold in *E*. *coli* HT115, while 22 genes were upregulated (between 2- and 20-fold) in *E*. *coli* OP50 (**Fig 2A**, **S1 Table**). Interestingly, 12 of the latter 22 genes encode proteins predicted to be localized to the mitochondria (**Table 1**).

**Table 1:** Genes with mitochondrial functions upregulated on *E*. *coli* OP50.

Gene	Name	Evidence
C05C10.3		Ortholog of human mitochondrial CoA transferase (NP_000427.1); in *C*. *elegans* mitochondrial proteome [52]
C55B7.4	*acdh-1*	Ortholog of human mitochondrial short chain specific acyl-CoA dehydrogenase (NP_001600.1); responds to propionate level in *C*. *elegans* mitochondria [6]; in *C*. *elegans* mitochondrial proteome [52]
F09E10.3	*dhs-25*	Ortholog of mitochondrial dehydrogenase (NP_055049.1) [53]; in *C*. *elegans* mitochondrial proteome [52]
F09F7.4	*hach-1*	Ortholog of human mitochondrial CoA hydrolase (NP_055177.2), in *C*. *elegans* mitochondrial proteome [52]
F22B8.7		Ortholog of human mitochondrial amidoxime reducing component (NP_073583.3), in *C*. *elegans* mitochondrial proteome [52]
F28F8.2	*acs-2*	Ortholog of human mitochondrial acyl-CoA synthetase (NP_079425.3), localized to mitochondria [54]; in *C*. *elegans* mitochondrial proteome [52]
F32D8.12		Ortholog of human mitochondrial lactate dehydrogenase (NP_705690.2); in *C*. *elegans* mitochondrial proteome [52]
F37B4.7	*folt-2*	In *C*. *elegans* mitochondrial proteome [52]
F44G3.2	*argk-1*	Ortholog of human mitochondrial creatine kinase (NP_001814)
F54D5.12		Ortholog of human mitochondrial hydroxyglutarate dehydrogenase (NP_689996.4), in *C*. *elegans* mitochondrial proteome [52]
Y22D7AL.5	*hsp-60*	Mitochondrial heat shock protein [55]; in *C*. *elegans* mitochondrial proteome [52]
Y38F1A.6	*hphd-1*	Ortholog of human mitochondrial hydroxyacid-oxoacid transhydrogenase (NP_653251.2), in *C*. *elegans* mitochondrial proteome [52]

This enrichment was strongly significant (*p* = 2.2*10^−16^), particularly given the small fraction of nuclear genes that encode mitochondrial proteins in *C*. *elegans* (~6%) and humans (7%) [52,56]. In contrast, only two genes encoding proteins targeted to the mitochondria (*acox-2* and T22B7.7) were upregulated in *E*. *coli* HT115 compared to *E*. *coli* OP50 (2/35 = 5.7%, *p* = 0.9896). Of the genes upregulated in worms fed *E*. *coli* OP50, two caught our attention: *hsp-60*, which encodes a mitochondrial chaperone upregulated on mitochondrial damage [57,58] and *acdh-1*, which encodes a short-chain acyl-CoA dehydrogenase that is upregulated when dietary sources are rich in branched-chain amino acids and/or propionyl-CoA, which can be toxic to mitochondria [59]. When these data were compared to a previous study, which used slightly different methodology to determine differentially regulated genes, the results had striking concordance (**S2 Table**). Overlap between the genes downregulated in each study or upregulated in each study was significant (*p*-value 1.42*10^−11^ and 4.97*10^−22^, respectively). Moreover, 70% of the genes downregulated in both studies are annotated as mitochondrially-localized or have a putative function in mitochondria.

We crossed the *acdh-1p*::GFP reporter into a *glp-4*(*bn2*) background, and then reared the resulting strain on either *E*. *coli* OP50 or *E*. *coli* HT115. We saw increased expression of *acdh-1p*::GFP in *E*. *coli* OP50-fed worms, whether measured by conventional imaging (**Fig 2B**, see **S7A Fig** for quantification) or by flow vermimetry (**Fig 2C**, see **S7B Fig** for quantification). Our transcriptome profiling and *acdh-1* expression data are in concordance with previous reports, where an *E*. *coli* OP50 diet increased expression of *acdh-1p*::GFP and transcription of several other genes encoding mitochondrially-targeted proteins [22,23]. These traits have been associated with a mild to moderate vitamin B12 deficiency [6,21].

We attempted to use mass spectrometry to quantify adenosylcobalamin in worms fed with either *E*. *coli* OP50 or *E*. *coli* HT115. We also tried to quantify it in bacterial slurries from each strain. Unfortunately, B12 levels for all samples measured were below the detection threshold of the instrument. Although we were unable to measure precise amounts of vitamin B12 in the host or in the bacteria, qualitative methods are available to test whether *E*. *coli* HT115 is a better source of vitamin B12, based on *acdh-1p*::GFP fluorescence and propionate toxicity. First, the bacterial growth media was spiked with exogenous methylcobalamin to a final concentration of 200 ng/mL, bacteria were grown as usual, and spotted onto NGM media plates. Worms were reared on these plates and then *acdh-1p*::GFP expression was measured at the young adult stage using flow vermimetry. Vitamin supplementation dramatically reduced *acdh-1p*::GFP expression (**Fig 2C, S7A Fig**). The same approach was used, with differing concentrations, to determine the amount of exogenous B12 required to reduce *acdh-1p*::GFP fluorescence in worms reared on each food. Comparable fluorescence was observed when *E*. *coli* OP50 and *E*. *coli* HT115 were supplemented with 50 μM and 3.1 μM, respectively (**Fig 2D and 2E**).

Second, expression of *acdh-1p*::GFP can be compared when worms are exposed to exogenous propionate [22,23]. Although worms reared on *E*. *coli* OP50 consistently showed more fluorescence than worms fed *E*. *coli* HT115, adding propionate to media increased *acdh-1p*::GFP expression regardless of diet (**Fig 2F and 2G**, see **S7B Fig** for quantification). For example, fluorescence was similar in worms reared on *E*. *coli* OP50 and *E*. *coli* HT115 spiked with 25 mM and 50 mM propionate, respectively.

Finally, in addition to triggering *acdh-1* expression, excess propionate is toxic to *C*. *elegans* [22,23,60]. Vitamin B12 sufficiency is proportional to increased survival. Young adult worms reared on either *E*. *coli* OP50 or *E*. *coli* HT115 were exposed to varying concentrations of propionate and survival was measured after 24h. Worms reared on *E*. *coli* HT115 exhibited increased resistance compared to *E*. *coli* OP50, and the addition of methylcobalamin to their diet improved each group further still (**Fig 2H**). All three assays qualitatively support the conclusion that *E*. *coli* HT115 is a better source of vitamin B12 than *E*. *coli* OP50, a finding that is consistent with previous reports [6,22,23].

### Vitamin B12 supplementation improves mitochondrial health

**Fig 3:**
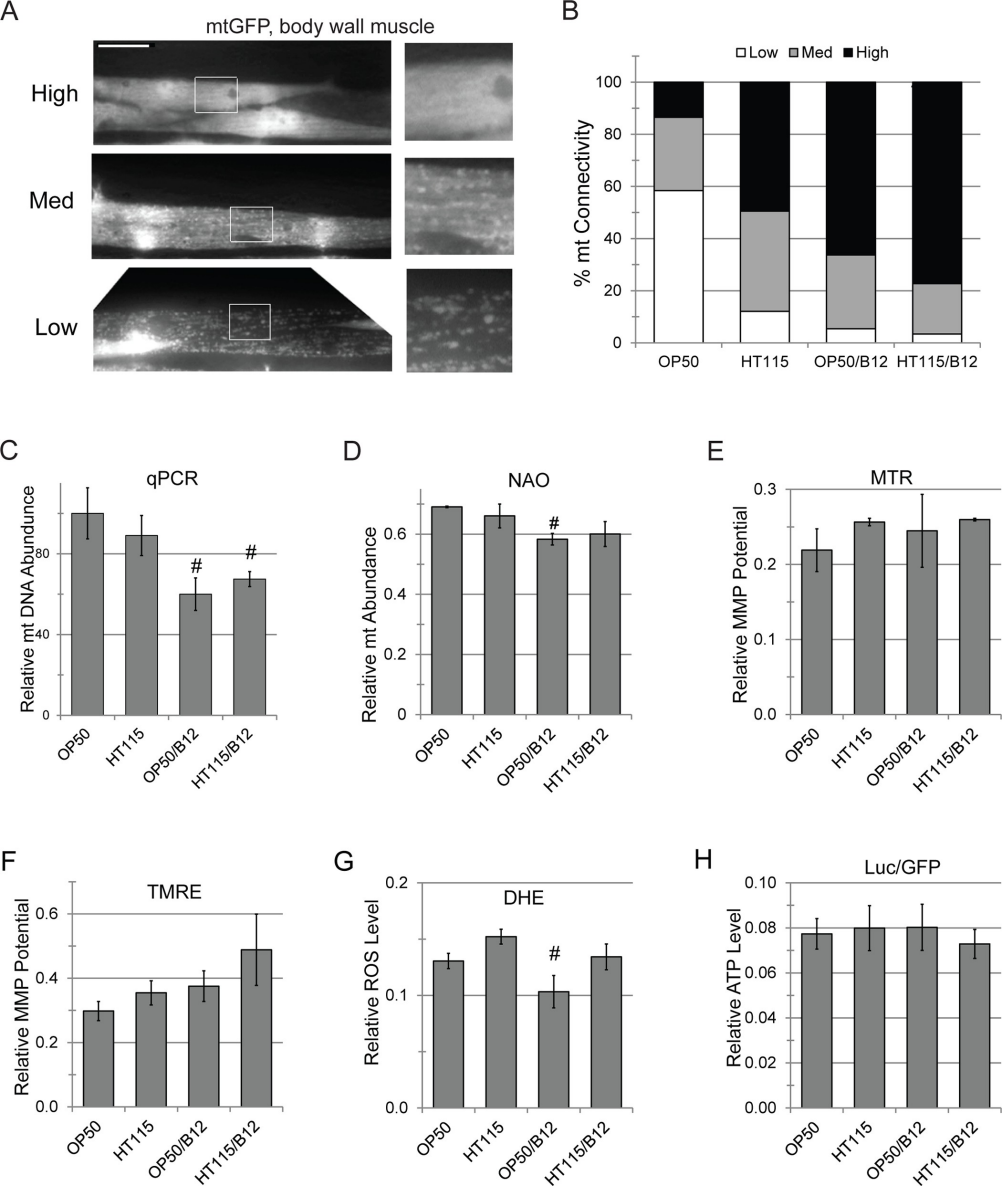
Methylcobalamin supplementation improves mitochondrial homeostasis. **(A)** *glp-4(bn2ts); zcIs14*|*myo-3*::*GFP(mt)* worms, encoding a mitochondrially-targeted GFP were fed *E*. *coli* OP50 or *E*. *coli* HT115 with or without methylcobalamin supplementation. Body wall muscle mitochondria were imaged in the same tail cell of worms from each condition. Worms were grouped into high, medium, or low connectivity, as shown by inset images. **(B)** Quantification of mitochondrial phenotypes from **(A)**. **(C)** Relative abundance of mitochondrial genome count in worms fed on *E*. *coli* OP50 or *E*. *coli* OP50 supplemented with methylcobalamin. **(D)** Measurement of mitochondrial membrane staining with nonyl-acridine orange (NAO). **(E,F)** Mitochondrial membrane potential, as determined by staining with MitoTracker Red **(E)** or tetramethylrhodamine ester (TMRE) **(F)**, in worms fed as in **(A)**. **(G)** Measurement of ROS production in worms, measured via fluorescence of dihydroethidium, fed as in **(A)**. **(H)** ATP production in worms fed as in **(A)**. Luminescence was normalized to GFP fluorescence to control for protein expression. *—*p*<0.05, *p*-value was calculated based on Student’s *t*-test. For **(B)** at least 30 worms were used per diet per biological replicate. For **(C-G)** at least 2,000 worms were used per biological replicate. For **(H)** at least 4 wells with 100 worms per well per condition per replicate. At least three biological replicates were performed for each experiment.

Because excess propionate is known to cause mitochondrial toxicity [59,61], we assayed mitochondrial structure in *C*. *elegans* fed either *E*. *coli* OP50 or *E*. *coli* HT115. Under normal conditions, mitochondrial quality control involves constant fission and fusion events that serve to pool healthy, functional mitochondrial content, while damaged material is sequestered for autophagic recycling [62]. A *C*. *elegans* strain expressing a mitochondrially-targeted GFP [57] was reared on *E*. *coli* OP50 or *E*. *coli* HT115 with or without methylcobalamin supplementation, and mitochondria were imaged in the same body wall muscle cell of young adult *C*. *elegans*. Worms fed *E*. *coli* HT115 or bacteria supplemented with methylcobalamin showed increased connectivity and fewer punctae than worms with less B12 (**Fig 3A and 3B**). The fragmentation induced by a diet of *E*. *coli* OP50 was greater than that observed when RNAi was used to knock down *fzo-1*/*MFN*, a mitofusin essential for proper mitochondrial fusion and homeostasis (**S8 Fig**).

Breakdown of the mitochondrial network is commonly associated with reduced organellar function. We assayed other metrics of mitochondrial health in worms reared on *E*. *coli* OP50 or *E*. *coli* HT115 with or without exogenous vitamin B12. Methylcobalamin supplementation slightly, but statistically significantly, decreased the average mitochondrial genome count as measured by quantitative PCR of mitochondrial to nuclear genome count (**Fig 3C**). Mitochondrial mass was slightly diminished when measured using nonyl-acridine orange staining (NAO, **Fig 3D**). Two different dyes to measure mitochondrial membrane potential (tetramethylrhodamine ethyl ester (TMRE) and mitotracker red (MTR)) showed no significant difference on any of the diets (**Fig 3E and 3F**), which suggested that B12 may slightly improve the average mitochondrial membrane potential. Supplementation with methylcobalamin reduced superoxide production as an indicator of ROS in worms fed OP50, but did not have a significant effect on worms reared on HT115 (p = 0.059) (**Fig 3G**). ATP production (as measured by luciferase activity) showed no significant difference after B12 supplementation (**Fig 3H**). Combined, these data indicate that B12 sufficiency correlates with an increase in mitochondrial connectivity and decreased mitochondrial number. This permits mitochondrial function to be carried out at a similar level with fewer mitochondria.

### Vitamin B12 supplementation increases stress resistance

**Fig 4:**
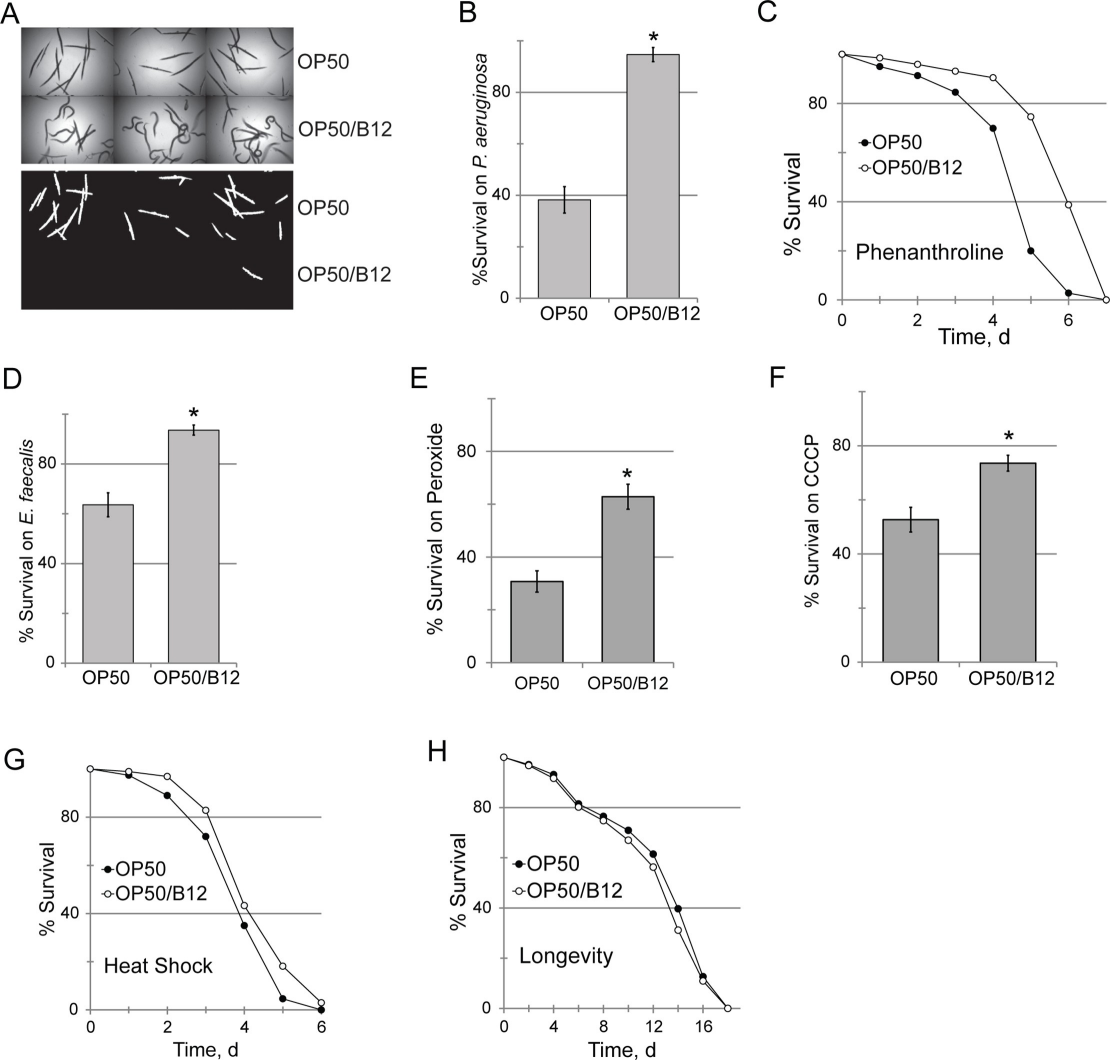
Methylcobalamin supplementation improves host resistance. **(A-B**) Representative images and quantification of survival of *glp-4 (bn2) C*. *elegans* exposed to *P*. *aeruginosa* PA14 after feeding on OP50 with or without methylcobalamin (B12) supplementation. **(C-F)** Effect of feeding on *E*. *coli* OP50 supplemented with methylcobalamin on *C*. *elegans* resistance to phenanthroline **(C)**, infection with *E*. *faecalis* **(D)**, peroxide exposure **(E)**, CCCP poisoning **(F)**, or hyperthermia **(G)**, or lifespan **(F).** *p*-value for **(E)** <0.01; **(F)** > 0.05 (N.S). *p-values* were calculated using log-rank test **(E, F)** or Student’s *t*-test **(B-D)**. *—*p*<0.01. For **(A, B, D, E, F)**, 10 wells, each containing 20 worms, were used for each diet for each replicate. For **(C, G, H)**, 50 worms were used per plate, three plates per replicate per condition. Three biological replicates were performed for each experiment.

We also tested whether methylcobalamin supplementation could alleviate the broad-spectrum sensitivity exhibited by *E*. *coli* OP50-fed worms. Worms were reared on *E*. *coli* OP50 grown with or without exogenous methylcobalamin, and their survival was tested in Liquid Killing. A dramatic difference was observed; less than half of OP50-fed worms remained alive by the time ~10% of OP50/B12-fed worms died (**Fig 4A and 4B**). Increased vitamin B12 availability also improved resistance to phenanthroline, infection with *E*. *faecalis*, peroxide stress, the mitochondrial poison carbonyl cyanide *m*-chlorophenyl hydrazone (CCCP), and hyperthermia (**Fig 4C–4G**). The resistance of wild-type worms fed on *E*. *coli* OP50 supplemented with methylcobalamin was also increased (**S9 Fig**).

A growing body of evidence suggests that decreasing mitochondrial activity, either by genetically compromising oxidative phosphorylation or by caloric restriction, extends lifespan [63,64]. Lifespan was unaffected by methylcobalamin supplementation (**Fig 4H**), as was seen when *E*. *coli* OP50 was replaced with *E*. *coli* HT115 (**Fig 1G**).

### The *E*. *coli* TonB transporter mediates bacterial vitamin B12 uptake

**Fig 5:**
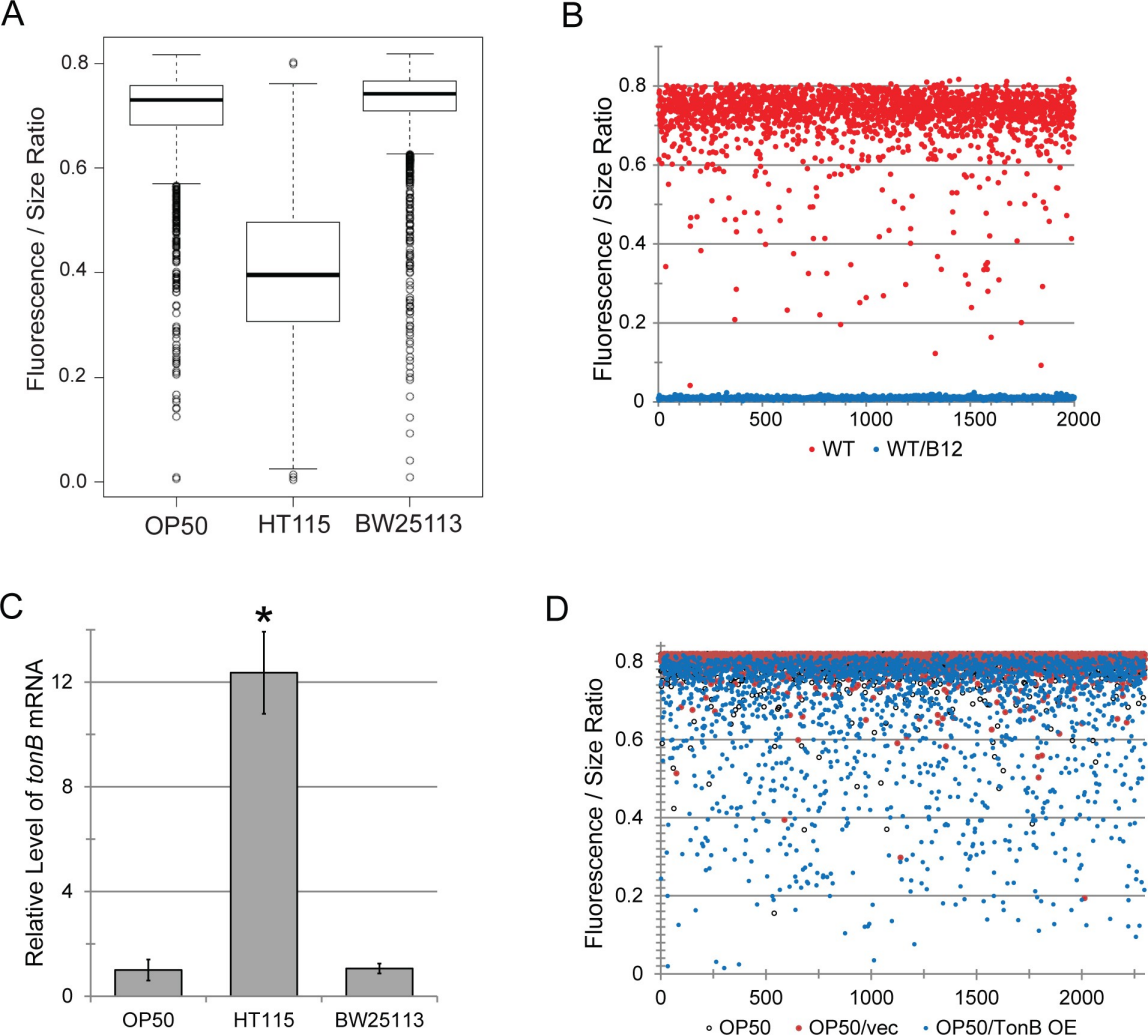
*acdh-1p*::GFP expression negatively correlates with TonB expression. **(A)** Box plot representation of basal *acdh-1*∷GFP expression in *E*. *coli* OP50, *E*. *coli* HT115, or *E*. *coli* BW25113. **(B)** Flow vermimetry measurements of the *acdh-1p*::GFP reporter fed with *E*. *coli* BW25113 with or without methylcobalamin (200 ng/mL) supplementation. **(C)** Relative level of *tonB* mRNA in panel of *E*. *coli* as in **(A)**. **(D)** Flow vermimetry measurements of the *acdh-1p*::GFP reporter fed with an *E*. *coli* OP50, OP50 + pBAD, or OP50 + pBAD-OPtonB (*tonB* overexpression). *—*p*<0.01 based on Student’s *t*-test. For **(A, B, D)**, at least 2,000 worms were used per condition per biological replicate. Three biological replicates were performed for each experiment.

All of these observations suggest that *E*. *coli* OP50 provides inadequate vitamin B12 for *C*. *elegans*. Since *E*. *coli* cannot synthesize vitamin B12, the bacterium must import it from the extracellular milieu, a process that has been reported to require TonB [65,66]. On this basis, we hypothesized that a bacterial strain lacking *tonB* would also trigger B12 deficiency in *C*. *elegans*. This prediction was tested with two different *tonB* deletions in *E*. *coli* BW25113 [67]. To establish a baseline, expression of *acdh-1p*::GFP was determined for *C*. *elegans* reared on *E*. *coli* BW25113. This diet largely recapitulated *acdh-1p*::GFP levels observed for *E*. *coli* OP50 (**Fig 5A**), indicating that this diet also likely causes a vitamin B12 deficiency. As with *E*. *coli* OP50 and *E*. *coli* HT115, supplementation of wild-type BW25113 with methylcobalamin completely abolished *acdh-1p*::GFP fluorescence (**Fig 5B**). qRT-PCR data on log phase growth cultures of each strain indicate that *tonB* levels for *E*. *coli* HT115 are approximately 12 times higher than *E*. *coli* OP50 or *E*. *coli* BW25113 (**Fig 5C**). Although *tonB* is important for the import of several large molecules, such as cobalamin, nickel complexes, and some carbohydrates [68], it is also required for infection by several bacteriophages [69], suggesting that there may be an evolutionary advantage in limiting its expression.

To determine whether this limitation in *tonB* expression could be overcome, the *tonB* locus was cloned into pBAD for inducible expression and the resulting construct was transformed into *E*. *coli* OP50. *tonB* expression was induced with 0.2% *L*-arabinose in LB media unsupplemented with methylcobalamin and then bacteria were spotted onto NGM. Worms were reared on this food source, and *acdh-1p*::GFP expression was assayed in young adults. A small, but statistically significant and repeatable reduction in *acdh-1p*::GFP fluorescence was seen (**Fig 5D**). It is unclear why the overexpression did not have a more pronounced effect. One possibility is that overexpression of TonB, which is present in the cytoplasmic membrane [70], prevents it from being properly folded and/or inserted into the membrane. Additionally, TonB is a member of a protein complex; altering the stoichiometry of proteins in complexes by overexpression often results in unpredictable consequences and poor expression, which has led to the 'balance hypothesis' [71–73]. Either, or both, of these phenomena may have limited the proper expression or function of TonB.

Surprisingly, neither *tonB* deletion allele completely blocked the attenuation of *acdh-1p*::GFP fluorescence seen from methylcobalamin supplementation (**S10 Fig**). This suggests that the bacteria may have an alternative, *tonB-*independent route to obtain cobalamin, although the process appears to be inefficient. Alternatively, the cells may be generating B12 *de novo* by salvaging precursors that they can convert into B12.

### B12-dependent resistance relies upon the methylmalonate-CoA/succinyl-CoA pathway of propionate breakdown

There are three obvious explanations for the vitamin B12-mediated resistance we observed. First, the additional vitamin B12 may alter the metabolism of *E*. *coli* (e.g., a deficiency of vitamin B12 in the LB broth used may limit the quality of the nutrition provided by the *E coli*, supplementation decreases the production of a toxic bacterial metabolite, or the bacteria may increase production of one or more salubrious compounds). Second, the bacterium may modify or metabolize the vitamin, creating the product that improves health. Finally, the bacteria may merely be the means of transporting the B12 into the intestine of *C*. *elegans*, where it directly alters host metabolism. To rule out the first two possibilities, we spotted heat-killed *E*. *coli* onto NGM plates that were themselves supplemented with methylcobalamin. *glp-4(bn2)* worms spotted onto these plates also exhibited increased resistance to *P*. *aeruginosa* Liquid Killing (**S11 Fig**).

**Fig 6:**
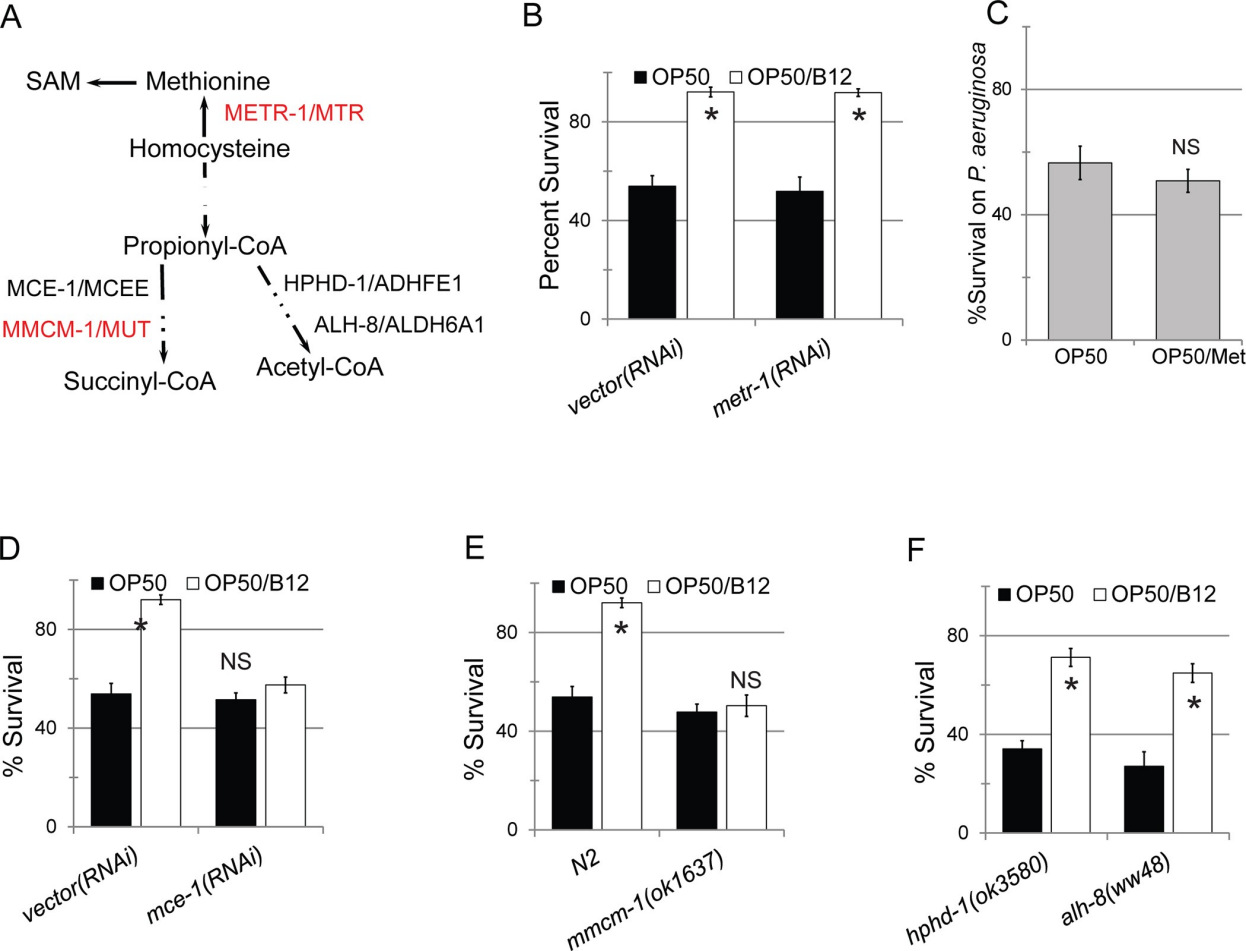
Rescue by methylcobalamin depends on methylmalonyl-CoA/succinyl-CoA clearance. **(A)** Schematics of biochemical pathways for methionine and propionyl-CoA metabolism in *C*. *elegans*. Proteins in red utilize B12 as a cofactor. **(B)** Survival of *C*. *elegans* fed on *E*. *coli* HT115 carrying empty vector or *metr-1(RNAi)* with or without exogenous methylcobalamin after exposure to *P*. *aeruginosa* PA14. **(C)** Survival of *C*. *elegans* reared on *E*. *coli* OP50 or OP50 supplemented with *L*-methionine after *P*. *aeruginosa* PA14 exposure. **(D-F)** Survival of wild-type *C*. *elegans* or mutants in two different propionate breakdown pathways with or without methylcobalamin supplementation after *P*. *aeruginosa* PA14 exposure. NS–*p*>0.05, *—*p*<0.01 based on Student’s *t*-test. For **(B, C, D, E, F)** 10 wells, each containing 20 worms, are used per condition per replicate. Three biological replicates were performed for each experiment.

On this basis, it was clear that *E*. *coli* is merely a vehicle for transporting vitamin B12 into *C*. *elegans*. As noted above, vitamin B12 functions as an essential co-factor for two enzymes: METR-1/MTR and MMCM-1/MUT (**Fig 6A**) [74]. To determine which of these functions underlies the improvement conferred by vitamin B12, survival in Liquid Killing was assessed in worms with mutations for each pathway.

RNAi targeting either *metr-1*/MTR or *mtrr-1*/MTRR had no effect on vitamin B12-mediated resistance to *P*. *aeruginosa* in the Liquid Killing assay (**Fig 6B**). To rule out the possibility of incomplete or insufficient RNAi knockdown, we also tested whether supplementing bacteria with 3 mM methionine (instead of methylcobalamin) improved host survival. Since this pathway uses vitamin B12 to generate methionine, supplementation with this amino acid should have the same result if this pathway is relevant. Consistent with the results from the *metr-1(RNAi)* and *mtrr-1(RNAi)* mutants, methionine supplementation had no impact on survival (**Fig 6C**). In contrast, disruption of the other B12-utilizing pathway abolished resistance even in the presence of exogenous methylcobalamin. RNAi targeting *mce-1*/MCEE or mutation of *mmcm-1*/MUT completely abolished the benefit from methylcobalamin supplementation (**Fig 6D and 6E**).

*C*. *elegans* maintains an alternative, B12-independent, pathway to metabolize propionyl-CoA. This shunt is more toxic and utilizes acrylyl-CoA and hydroxypropionyl-CoA intermediates [23]. We also tested whether this pathway plays a role in mitochondrial toxicity of dietary B12 deficiency. Mutations in *hphd-1*/ADHFE1 or *alh-8*/ALDH6A1, two genes in this shunt, had no effect on the ability of vitamin B12 supplementation to promote survival (**Fig 6F**). This further supports our conclusion that the MMCM-1/MUT pathway is the relevant target for B12-mediated improvements in host health.

## Discussion

While probing the mechanisms of pathogenesis of *P*. *aeruginosa* in *C*. *elegans*, we discovered that switching between two commonly used strains of *E*. *coli*, OP50 and HT115, dramatically altered host stress tolerance. Further investigation linked these differences to improved mitochondrial homeostasis in worms reared on *E*. *coli* HT115 and suggest that this is likely due to the buildup of propionyl-CoA, a mitotoxic byproduct of branched chain amino acid metabolism, in worms reared on *E*. *coli* OP50.

Normally, propionyl-CoA is converted to succinyl-CoA, via a methylmalonyl-CoA intermediate, in a vitamin B12-dependent fashion. However, several lines of evidence, as well as previous reports, suggest that a diet of *E*. *coli* OP50 generates a vitamin B12 deficiency in the host. It is worth noting that this deficiency is likely to be relatively mild, since severe vitamin B12 deficiency causes drastic phenotypes in *C*. *elegans*, including infertility, growth retardation, and shortened lifespan [20]. However, we saw no gross differences in growth rate or lifespan in worms when they were provided with *E*. *coli* HT115 or *E*. *coli* OP50 supplemented with methylcobalamin as compared to *E*. *coli* OP50 alone.

Several lines of evidence support our conclusion that *E*. *coli* HT115 is a better source of vitamin B12 than *E*. *coli* OP50. For example, worms reared on *E*. *coli* HT115 had diminished *acdh-1p*::GFP fluorescence and decreased propionate toxicity. Moreover, supplementation of an OP50 diet with methylcobalamin conferred HT115-like characteristics in terms of mitochondrial homeostasis and vitamin B12 phenotypes. Tellingly, RNAi targeting *mce-1*/MCEE or *mmcm-1*/MUT mutations abolished the health improvement conferred by methylcobalamin supplementation, indicating that this pathway and this metabolite were involved. Moreover, worms reared on *E*. *coli* OP50 grown in minimal media showed delays in egg-laying compared to worms fed *E*. *coli* OP50 supplemented with vitamin B12 or *E*. *coli* HT115 (**S12 Fig**). Worms serially passaged on foods grown in this media also showed a substantial reduction in fecundity after 5 generations. Both of these findings are consistent with previous reports [20,23] and are indicative of acute and chronic vitamin B12 deficiency. Finally, elegant work by the Walhout group has indicated a vitamin B12 deficiency in OP50-reared worms [22].

### Increased pathogen sensitivity from B12 deficiency may be evolutionarily conserved

While malnutrition is well-known to predispose animals to infection, subtle nutritional defects, and their concomitant effects on host physiology, are more difficult to detect. Interestingly, our findings may explain an intriguing finding from a recently published study [75]. *Gif* mutant mice, which have compromised intestinal absorption of dietary B12, showed a striking increase in sensitivity and mortality to infection with *Salmonella enterica* serovar Typhimurium or *Citrobacter rodentium*. Although the infected mice showed no overt immune deficiencies, they had severe metabolic defects that included compromised mitochondrial function and dramatic increases in methylmalonate concentration. Importantly, these phenotypes were resolved by supplementation of the animals' diet with cobalamin. The authors postulated that the increased mortality may arise from metabolic starvation due to competition for nutrients between the host and its microbiota. However, their results can also be explained by our model. I.e., the mitochondrial damage inflicted by vitamin B12-deficiency made the host more sensitive to stress and pathogens and significantly increased mortality. Although published analyses are limited, there is also some evidence to suggest people with genetic priopionate acidemias, which similarly cause propionyl-CoA accumulation, may also be predisposed to bacterial and fungal infections [76].

### A consistent, defined diet is needed for *C*. *elegans*

Although we began our queries of the host-microbiota-diet axis with a minimal, binary system (*C*. *elegans* and *E*. *coli*), unexpected complexity has arisen. Differences in the biology of *C*. *elegans* reared on OP50 or HT115 have generally been attributed to the differences in the origins of the bacterial strains (i.e., OP50 was derived from an *E*. *coli* B strain while HT115 came from *E*. *coli* K12) or to their differences in nutrient composition (HT115 contains less triacylglycerols and pyrimidines, and more carbohydrates than OP50 [8,77] and increases host concentrations of some amino acids and other metabolites [7]). Our data suggest that *acdh-1p*::GFP expression, a marker for B12 deficiency, poorly correlates with strain origin. *E*. *coli* BW25113, which also originated from K12, showed *acdh-1p*::GFP fluorescence similar to OP50 (**Fig 5A**)).

Metabolic differences of this sort likely contribute substantial uncertainty to research outcomes, particularly since some labs use *E*. *coli* OP50 as the standard food, some use *E*. *coli* HT115, and others routinely use *E*. *coli* HB101. For example, despite the fact that the *C*. *elegans* literature is replete with comparisons of the lifespan of *C*. *elegans* reared on OP50 vs. HT115, no consensus conclusion has been reached. Some researchers have seen no difference in lifespan [8,45], others find an HT115-mediated extension of lifespan [9,78], and still others find OP50-reared worms surviving longer [6]. Explanations for these divergences abound, including variations in media composition (or even batch-to-batch variations from a single supplier), methodological differences in assays, or even accumulated mutations in the "wild-type" *E*. *coli* or *C*. *elegans* strains as they are propagated within and between labs.

Ultimately, the inability to resolve what should be a simple question (i.e., which food allows *C*. *elegans* to live longer?) is troubling, particularly since the phenotype being assayed (i.e., death) should be unambiguous and the experimental setup is straightforward. More elusive phenotypes with perhaps smaller effects and more complex experimental design likely have greater variability. Curran and colleagues [79] have persuasively argued the need for a rigorously-defined, standardized growth medium for studies of aging in *C*. *elegans*; our data suggest this need probably extends to all studies using this model.

## Materials and methods

### *C*. *elegans* strains

All *C*. *elegans* strains were maintained on nematode growth medium (NGM) seeded with *Escherichia coli* strain OP50, *E*. *coli* strain HT115, or *E*. *coli* BW25113 (see below). In some experiments, *E*. *coli* growth media were supplemented with methylcobalamin to a final concentration of 0.2 mg/L. Unless otherwise noted, worms were reared and passaged at 15°C [80], with the exception of *glp-4(bn2)* worms, which were sterilized prior to use by plating diapaused, synchronized L1 larvae on concentrated *E*. *coli* on NGM plates and kept at 25°C. For RNAi-mediated gene knockdown, plasmids from the Ahringer library [26] were either used in the *E*. *coli* HT115(DE3) strain supplied or were purified and transformed into *E*. *coli* OP50(*xu363*), an RNAi-competent strain of OP50 [45]. All plasmids were sequence verified.

*C*. *elegans* strains used in this study included N2 Bristol (wild-type), SS104 [*glp-4*(*bn2ts*)] [47], NVK44 [*glp-4(bn2ts); zcIs14*|*myo-3*::*GFP(mt)*|], PE327 (ATP reporter): *glp-4(bn2ts); feIs5* [*sur-5p*::*luciferase*::*GFP* + *rol-6(su1006)*] [81], RB1434[*mmcm-1*(*ok1637*)], RB2572[*hphd-1*(*ok3580*)], VL1176[*alh-8*(*ww48*)], and VL749 [*wwIs24*|*acdh-1*p::*GFP* + *unc-119(+)*|] [6].

### Bacterial strains

Bacterial strains used in this study included *E*. *coli* OP50, *E*. *coli* OP50(*xu363*) [45], *E*. *coli* HT115(DE3), and *E*. *coli* BW25113. Two *E*. *coli tonB* deletion alleles (JW5195-1 and JW5195-2) were taken from the Keio Knockout Collection (GE Dharmacon). To generate the *tonB* overexpression strain, genomic DNA was purified from *E*. *coli* strain OP50 used to amplify the *tonB* locus. The resultant PCR product was digested with *Nde*I and *Sal*I and subcloned into pBAD33.1 (a gift from Christian Raetz, Addgene plasmid #36267 [82]). The resultant plasmid, pBAD-OP*tonB* was transformed into chemically competent *E*. *coli* OP50 using conventional techniques. For pathogenesis assays, *P*. *aeruginosa* strain PA14 [83], *P*. *aeruginosa* PA14 tranformed to express DsRed [24], or *E*. *faecalis* strain OG1RF [84] were used.

### Heat-Killed *E*. *coli* OP50

To prepare heat-killed *E*. *coli* OP50, *E*. *coli* from an overnight culture in LB were centrifuged and concentrated tenfold in S Basal. Bacteria were then killed by incubation at 75°C for 30 min with regular mixing by inversion. Absence of live *E*. *coli* in each preparation was confirmed by plating on LB agar.

For use in Liquid Killing assays, 2 mL of concentrated dead bacteria were plated onto modified NGM that was supplemented with vitamin B2 to a final concentration of 0.3 mg/L. Normally, *C*. *elegans* grows poorly on dead *E*. *coli*. However, exogenous vitamin B2 significantly improves *C*. *elegans* health under these conditions [85]. To test vitamin B12 supplementation, the NGM also supplemented with 0.2 mg/L methylcobalamin.

### *C*. *elegans* pathogenesis, chemical exposure, hyperthermia, and longevity assays

Liquid Killing assays with *P*. *aeruginosa* PA14 were performed as described [25]. The *C*. *elegans*—*E*. *faecalis* assay was performed as described [31]. Assays were timed such that ~60–70% worms reared on *E*. *coli* OP50 would be dead. At least three biological replicates were performed per condition per experiment. Each biological replicate contained at least 10 wells (~200 animals). Statistical significance was determined using Student’s *t*-test.

For the juglone survival assay, *glp-4(bn2*) worms were grown to young adulthood and placed on NGM agar media supplemented with 120 μM of juglone [86]. For peroxide and carbonyl cyanide *m*-chlorophenyl hydrazone (CCCP) exposure worms were treated with 2 mM or 100 μM of drug in S Basal, respectively. Survival was scored using Sytox Orange stain after 20 h of incubation. Phenanthroline survival assays were done similarly, except plates were supplemented with 100 μM of this chelator. In each case, plates were made fresh and used the same day. Hyperthermia assays were performed at 30°C. In all cases, unsupplemented NGM agar plates incubated at 25°C were used as controls. Worms were scored daily and worms were scored as dead when they failed to respond to touch. Longevity assays were performed similarly, except worms were incubated at 25°C on the appropriate food source for the duration of the experiment and scored every other day. Worms were considered dead when they failed to respond to a gentle prod to the head. At least three biological replicates were performed for each experiment. Each biological replicate consisted of three plates with ~50 worms/plate. Statistical significance was determined using log-rank test (http://bioinf.wehi.edu.au/software/russell/logrank/). Worms on plates were censored if they left the agar plate.

The propionate sensitivity assay was performed as described in [22] with modifications. 70 L1-stage worms were dropped on to NGM agar plates containing appropriate concentrations of propionate (or no propionate control) using a COPAS FlowSort. After 72 h incubation at 25°C, adult worms were counted and survival was calculated. At least three biological replicates were performed for each experiment. Statistical significance was determined using Student’s *t*-test.

### Detection of total iron via ICP-MS

In brief, approximately 24,000 *C*. *elegans* L1 larvae (per sample) were raised on NGM plates seeded with *E*. *coli* OP50 or *E*. *coli* HT115. Upon reaching young adulthood, worms were transferred to 15 mL conicals, washed four times, and then collected with identical volumes. Samples were frozen at -80°C and processed as described [29]. At least three replicates were tested for each diet. Significance was tested using Student's *t*-test.

### Detection of host ferric iron

*glp-4(bn2)* L1 larvae were spotted onto NGM plates seeded with either *E*. *coli* OP50 or *E*. *coli* HT115 and allowed to develop to young adulthood at 25°C. Worms were collected, lysed, and fluorometric determination of ferric iron was performed as previously described [29].

### Microarray

For RNA collection, *glp-4(bn2ts)* worms were grown on appropriate plates until reaching young adult stage. Three biological replicates were performed. RNA was purified using Trizol following by cleanup according to Qiagen protocol. cDNA and cRNA were synthesized and hybridized to Affymetrix GeneChips for *C*. *elegans* at the Partners Center for Personalized Genetic Medicine, Boston, MA, according to manufacturer’s protocols. Three biological replicates were tested for each condition. Gene expression was analyzed using GCRMA (http://www.bioconductor.org). Differentially regulated genes were determined on the basis of fold change (>2) and the value of a modified Wilcoxon rank test (>1.5). The Wilcoxon coefficient was determined for each probeset as the smallest expression value in the condition with higher average divided by the highest expression value in the condition with lower average. Microarray data were deposited in GEO database and are available using following link: https://www.ncbi.nlm.nih.gov/geo/query/acc.cgi?token=kjibcyuydrstvuj&acc=GSE97678

### Quantitative Real-Time PCR (qRT-PCR)

For determination of the basal level of immune genes in *C*. *elegans*, ~10,000 of OP50- and HT115-fed *glp-4(bn2ts)* worms were grown to young adult stage and RNA was purified as described above. cDNA synthesis was performed according to manufacturers’ protocols (Ambion). qRT-PCR was performed using SYBR Green iQ mix (Bio-Rad). *snb-1* gene was used as a control. For each gene, threshold cycle (Ct) was determined and ΔCt from *snb-1* was calculated.

For measurement of relative abundance of mitochondrial DNA, total DNA was extracted from ~2,000 synchronized young adult worms grown on appropriate food source. For genome comparisons, *nd1* was used as a mitochondrial gene and *act-3* as nuclear, as previously described [87]. Resultant DNA was used for to perform quantitative PCR using SYBR Green (BioRad), and mitochondrial gene level was normalized to nuclear gene number using the ΔΔCt method. Thermocycler parameters were as for qRT-PCR.

For measurements of *tonB* expression in *E*. *coli* strains, RNA was purified as described above, except samples were treated with DNAase I (NEB). *cysG* and *idnT* were used as housekeeping genes [88]. Fold changes were calculated using ΔΔCt method.

Primer sequences are available upon request. Three biological replicates were performed per test, each biological replicate contained duplicate wells for each primer/cDNA combination. Statistical significance was calculated using Student’s *t*-test.

### Imaging

For visualization of *myo-3*::GFP(mt) and *acdh-1p*::GFP on slides, L1 larvae were spotted onto NGM plates seeded with appropriate bacteria, and allowed to mature into young adults at 25°C. Worms were immobilized with levamisole and transferred to slides. Images were acquired using a Zeiss Axio Imager M2 upright microscope with a Zeiss AxioCam 506 Mono camera and Zeiss Zen2Pro software. All fluorescent images were collected under identical exposure conditions. Fluorescence quantification was based on at least 50 worms per condition per biological replicate. At least three biological replicates were performed. Statistical significance was determined by Student’s *t*-test.

### ATP determination in live worms

For determination of ATP concentrations, approximately 2,000 PE327 L1 larvae [64] were placed onto NGM plates seeded with either *E*. *coli* OP50 or *E*. *coli* HT115 and allowed to develop to young adult stage at 25°C. Worms were then transferred to 96-well, white clear bottom plates and washed with S-basal five times. 150 μL Luminescence buffer (0.14M K2PO4, 0.03M sodium citrate, 1% DMSO, 1mM luciferin) was added to each well in the plate. Bioluminescence and GFP fluorescence were measured after 30 min using a Cytation5 multimode plate reader/imager (BioTek). Luminescence values were normalized to GFP fluorescence to control for differences in protein expression.

### Flow vermimetry

For measurement of *acdh-1p*::GFP, 5,000 VL749 L1 larvae were spotted onto NGM plates seeded with the food sources described. In some cases, NGM was supplemented with propionate and/or methylcobalamin. Worms were collected into 50 mL conicals, washed three times, and then GFP fluorescence was measured using a COPAS FlowPilot with 488 nm excitation and a long-pass filter.

Mitochondrial membrane potential and reactive oxygen species were measured in a similar fashion, except that *glp-4(bn2)* worms were used, and worms were stained as described for MitoTracker Red [28]. nonyl-acridine orange (NAO) and tetramethylrhodamine ester (TMRE) dyes were used to stain mitochondria at 10 μM and 5 μM, respectively. ROS measurements were performed similarly, except dihydroethydium at a concentration of 3 μM and excitation at 488 nm was used. In each case, worms were stained for 1h, then washed 5 times prior to being measured. Measurements for each stain/diet combination were taken with the same settings. At least 1,500 worms/condition/biological replicate were used; at least three biological replicates were performed. Statistical significance was determined by Student’s *t*-test.

### Fertility assessment

Worm fecundity was scored as described by Bito *et al*., [20] with minor modifications. *E*. *coli* OP50, HT115, and OP50 supplemented with 100 μM methylcobalamin were grown for 3 days in M9 medium at 37°C, then concentrated and plated onto normal NGM. Single *glp-4(bn2)* larvae were transferred to a new plate containing the same food. After reaching late-L4 stage, worms were allowed to lay eggs for 24 h. Young adult worms were then transferred to a fresh plate with the same food and allowed to complete egg laying. After 48 h, progeny on both plates were counted (providing both the 24h count and the total count). One larva, chosen arbitrarily, was transferred to a new plate, to yield progeny for the subsequent generation. At least 24 plates were used per strain per biological replicates per generation; three biological replicates were performed. Statistical significance was determined by Student’s *t*-test.

## Supporting information

S1 Fig *E. coli* OP50 and *E. coli* HT115-fed *C. elegans* have similar iron content. **(A)** The presence of the empty pL4440 vector did not affect survival in Liquid Killing. **(B)** Relative abundance of ferric iron in worms fed *E*. *coli* OP50 or *E*. *coli* HT115. **(C)** ICP-MS analysis of total iron concentration in worms fed *E*. *coli* OP50 or *E*. *coli* HT115. N.S.—*p* > 0.05. based on Student’s *t*-test. For **(A)** 10 wells, each containing 20 worms, were used per condition per replicate. For **(B)**, approximately 18,000 worms were used per biological replicate per condition. For **(C)**, approximately 24,000 worms were used per biological replicate per condition. Three biological replicates were performed for each experiment.
(PDF)

S2 Fig *E. coli* HT115 does not affect *P. aeruginosa* slow killing. **(A)** *glp-4(bn2)* worms were reared on *E*. *coli* OP50 or *E*. *coli* HT115 and then used for *P*. *aeruginosa* PA14 slow-killing. **(B-C)** Fluorescent images **(B)** or quantification of fluorescence **(C)** of colonization by *P*. *aeruginosa* PA14-DsRed during slow kill assays after being reared on OP50 or HT115. Statistical significance was calculated based on log-rank test **(A)** or Student’s *t*-test **(C)**. No significant changes were observed. For **(A)**, 50 worms per plate, three plates per condition per replicate were used. For **(B)**, 30 worms per plate, three plates per condition per replicate were used. Three biological replicates were performed for each experiment.
(PDF)

S3 Fig A diet of *E. coli* HT115 does not change basal levels of expression of innate immune or stress pathway effector genes. **(A-E)** qRT-PCR analysis of gene expression levels for downstream effectors from the indicated innate immune or stress response pathway. *p*>0.05 for all primers tested. Statistical significance was calculated based on Student’s *t*-test. No significant changes were observed. About 10,000 worms were used per biological replicate per condition. Three biological replicates were performed for each experiment.
(PDF)

S4 Fig Worms fed *E. coli* OP50(*xu363*) and *E. coli* HT115 have similar sensitivity to RNAi. Starting at the L4 stage, *C*. *elegans* were fed RNAi-competent strains of *E*. *coli* OP50(*xu363*) or *E*. *coli* HT115 bacteria that contained plasmids driving expression of *cyc-1* RNAi constructs (which results in embryonic lethality). Each worm was placed onto an individual plate and moved to fresh plate daily. NS–*p*>0.05 based on Student’s *t*-test. At least 30 adults were used per biological replicate per condition. Three biological replicates were performed.
(PDF)

S5 Fig *E. coli* HT115-mediated resistance is not dependent upon activation of canonical innate immune or stress response pathways. **(A-E)** Starting at the L1 stage, *C*. *elegans* were fed *E*. *coli* OP50 or *E*. *coli* HT115 bacteria that contained plasmids driving expression of RNAi constructs that targeted innate immune or stress response genes. Young adult worms were subsequently exposed to *P*. *aeruginosa* PA14 for an appropriate length of time. Percent survival was inferred based on staining with Sytox Orange, a cell impermeant dye. *—*p*<0.01 based on Student’s *t*-test. 10 wells, 20 worms per well, were used per condition per biological replicate. Three biological replicates were performed for each experiment.
(PDF)

S6 Fig Methylcobalamin supplementation improves survival of wild-type worms. **(A-C)** Survival of wild-type (N2) *C*. *elegans* worms fed *E*. *coli* OP50 alone or supplemented with methylcobalamin or *E*. *coli* HT115-fed worms in *P*. *aeruginosa*-mediated Liquid Killing **(A)**, propionate intoxication **(B)**, or hyperthermia at 30°C **(C). (D)** Longevity of worms reared as in **(A)**. *—p< 0.01, based on Student’s *t*-test. For **(C, D)** *p*<0.01 was calculated using a log-rank test. For **(A),** ten wells, with 20 worms per well, were used for each condition for each replicate. For **(B-D)**, 70 **(B)** or 50 **(C, D)** worms per plate, three plates per condition were used per replicate. Three biological replicates were performed for each of the experiments.
(PDF)

S7 Fig Quantification of *acdh-1p::*GFP expression. **(A)** Quantification of *acdh-1p*::GFP reporter fluorescence in worms fed either *E*. *coli* OP50 or *E*. *coli* HT115, with or without methylcobalamin supplementation to a final concentration of 200 ng/ml, as indicated. **(B)** Quantification of *acdh-1p*::GFP expression in worms fed *E*. *coli* OP50 or *E*. *coli* HT115, with or without methylcobalamin supplementation to a final concentration of 200 ng/ml and exposed to propionate, using flow vermimetry. Fluorescence for each worm was normalized to its size. *—*p*<0.01 based on Student’s *t*-test. For **(A)** 30 worms per condition per replicate were measured. For **(B),** 2,000 worms were used per biological replicate per condition, a representative replicate is shown. Three biological replicates were performed for each experiment.
(PDF)

S8 Fig *Fzo-1/Mfn1(RNAi)* induces mitochondrial fragmentation. *glp-4(bn2ts); myo-3*::*GFP(mt)* worms were reared on either *E*. *coli* HT115 containing either an empty RNAi vector or an RNAi vector targeting *fzo-1/Mfn1* and then imaged to evaluate fragmentation of the mitochondrial network. Fragmentation was assessed as in **Fig 3**. 30 worms were used for each condition for each replicate. Three biological replicates were performed for each experiment.
(PDF)

S9 Fig Methylcobalamin supplementation improves resistance to mitochondrial poisons in wild-type worms. Wild-type (N2) worms were reared on *E*. *coli* OP50 with or without methylcobalamin supplementation and then exposed to toxic levels of either peroxide **(A)** or CCCP **(B)**. 10 wells, with 20 worms per well, were used for each condition for each replicate. At least 2 biological replicates were performed for each experiment. *—*p*<0.01 based on Student’s *t*-test.
(PDF)

S10 Fig *E. coli* BW25113 has a *tonB*-independent method for vitamin B12 import. *achd-1p*::GFP was measured via flow vermimetry in worms reared on two different *tonB* deletion strains in the *E*. *coli* BW25113 background sourced from the Keio collection. Bacteria were grown with and without exogenous methylcobalamin.
(PDF)

S11 Fig Methylcobalamin supplementation improves survival independently of bacteria. Survival of *C*. *elegans* reared on heat-killed *E*. *coli* OP50. NGM agar was either supplemented or not with 200 ng/mL of methylcobalamin. Worms were exposed to *P*. *aeruginosa* PA14 and survival was assessed after 42 h.
(PDF)

S12 Fig Bacterial media deficiency causes long-term changes to *C. elegans* fecundity. 24h and total brood sizes were determined for wild-type (N2) worms reared on *E. coli* OP50 with or without exogenous methylcobalamin, or *E*. *coli* HT115 that was grown in minimal M9 media. L4 larvae were transferred to fresh plates with the same food, allowed to lay for 24h and then were transferred to a new plate for another 24h. Fecundity was assessed by counting the number of hatched larvae. For each condition, 30 worms were tested per replicate per generation; at least three biological replicates were performed.
(PDF)

S1 Table List of genes differentially regulated by feeding on *E. coli* HT115 vs. *E. coli* OP50. Genes differentially regulated in young adult *glp-4(bn2)* worms, grown on *E*. *coli* HT115 since L1 stage. Fold changes shown are normalized to the genes’ expression levels in *E*. *coli* OP50.
(XLSX)

S2 Table Common genes between two transcriptome profiling studies on the effect of HT115 on *C. elegans* transcriptome. Microarray data on genes that were up- or down-regulated when worms were fed *E*. *coli* HT115 (as compared to the standard laboratory diet of *E*. *coli* OP50) were compared between [6] and this study.
(DOCX)
