# Supplementary figures and images for: Interplay between mitochondria and diet mediates pathogen and stress resistance in Caenorhabditis elegans
Source: PLoS Genet. 2019 Mar 13;15(3):e1008011. doi: 10.1371/journal.pgen.1008011 (PMC6415812; doi:10.1371/journal.pgen.1008011)

**A**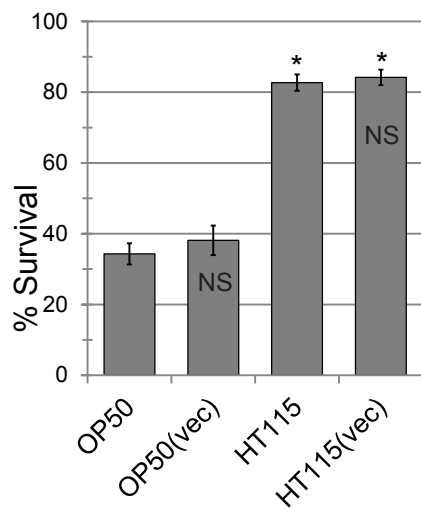**B**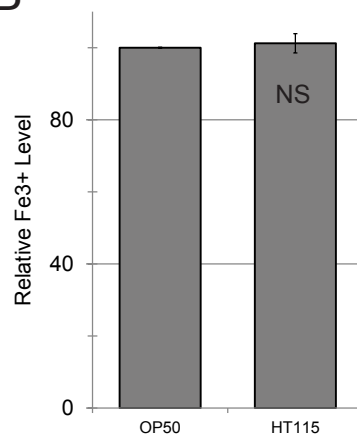**C**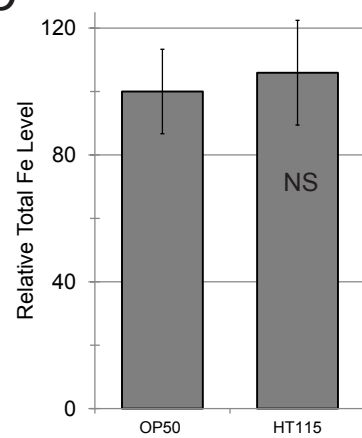

Figure S1

Supplement: S1 Fig — (A) The presence of the empty pL4440 vector did not affect survival in Liquid Killing. (B) Relative abundance of ferric iron in worms fed E. coli OP50 or E. coli HT115. (C) ICP-MS analysis of total iron concentration in worms fed E. coli OP50 or E. coli HT115. N.S.—p > 0.05. based on Student’s t-test. For (A) 10 wells, each containing 20 worms, were used per condition per replicate. For (B), approximately 18,000 worms were used per biological replicate per condition. For (C), approximately 24,000 worms were used per biological replicate per condition. Three biological replicates were performed for each experiment. (PDF) [file pgen.1008011.s001.pdf]

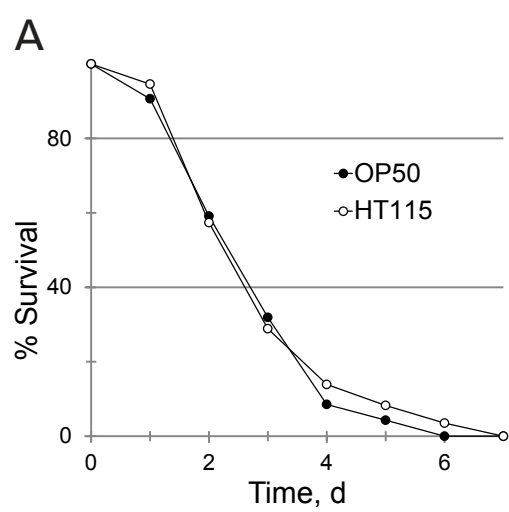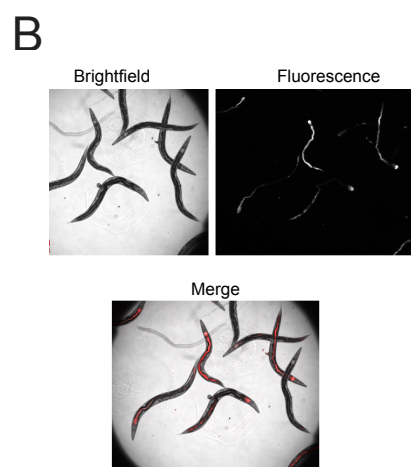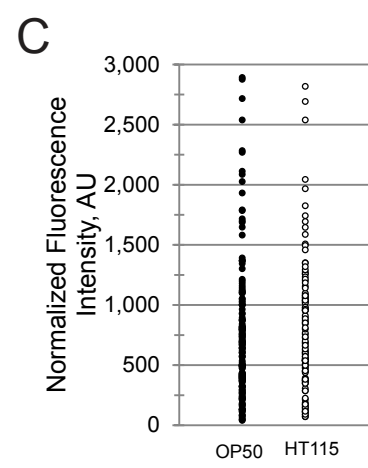

Figure S2

Supplement: S2 Fig — (A) glp-4(bn2) worms were reared on E. coli OP50 or E. coli HT115 and then used for P. aeruginosa PA14 slow-killing. (B-C) Fluorescent images (B) or quantification of fluorescence (C) of colonization by P. aeruginosa PA14-DsRed during slow kill assays after being reared on OP50 or HT115. Statistical significance was calculated based on log-rank test (A) or Student’s t-test (C). No significant changes were observed. For (A), 50 worms per plate, three plates per condition per replicate were used. For (B), 30 worms per plate, three plates per condition per replicate were used. Three biological replicates were performed for each experiment. (PDF) [file pgen.1008011.s002.pdf]

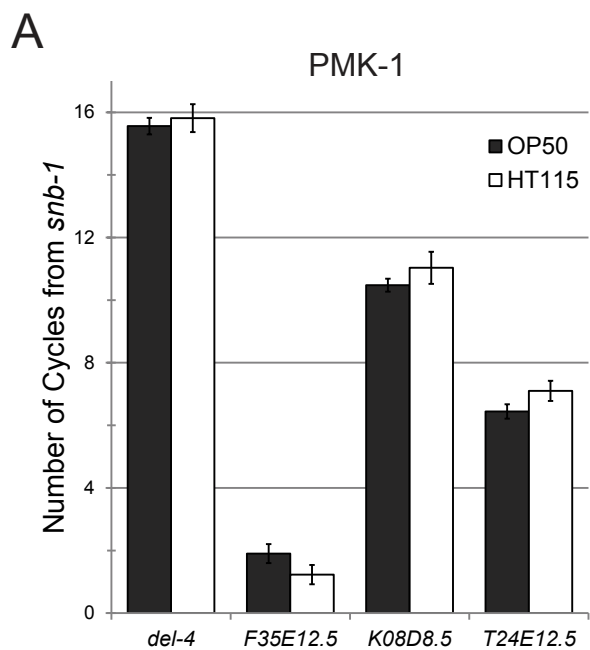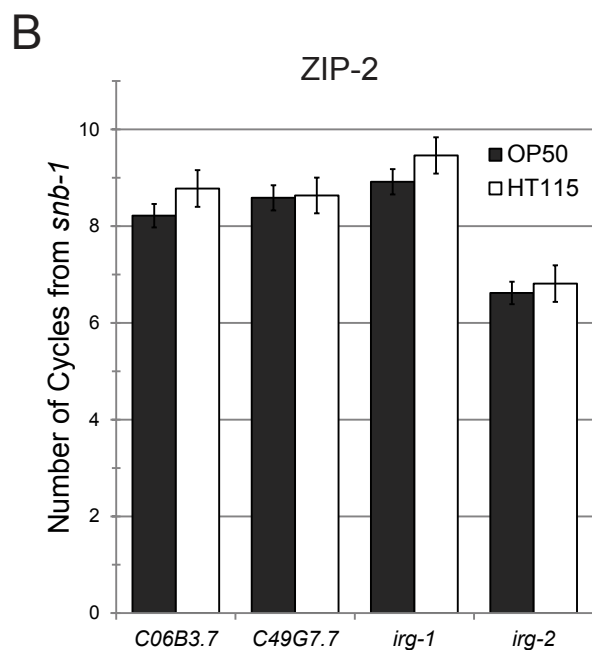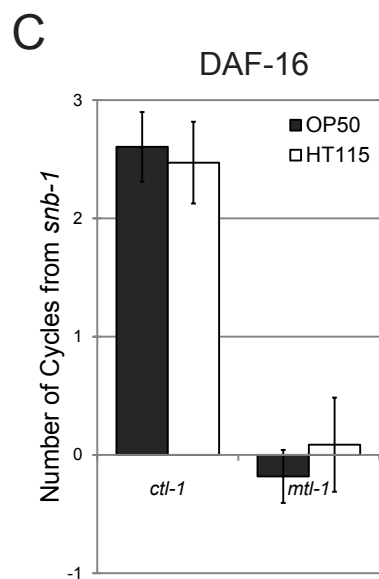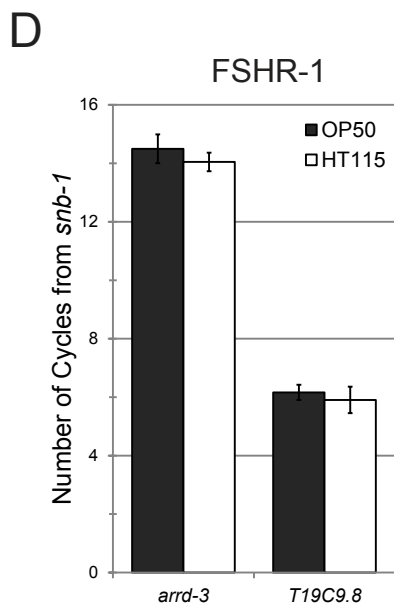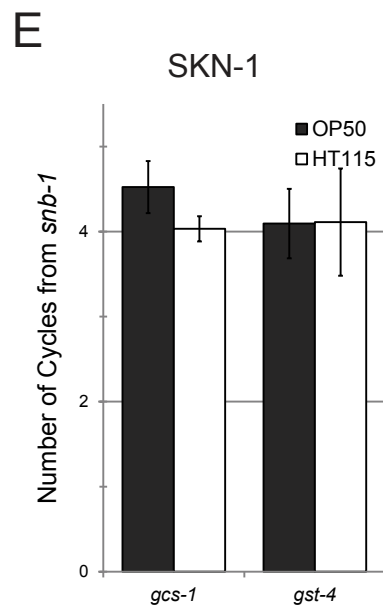

Figure S3

Supplement: S3 Fig — (A-E) qRT-PCR analysis of gene expression levels for downstream effectors from the indicated innate immune or stress response pathway. p>0.05 for all primers tested. Statistical significance was calculated based on Student’s t-test. No significant changes were observed. About 10,000 worms were used per biological replicate per condition. Three biological replicates were performed for each experiment. (PDF) [file pgen.1008011.s003.pdf]

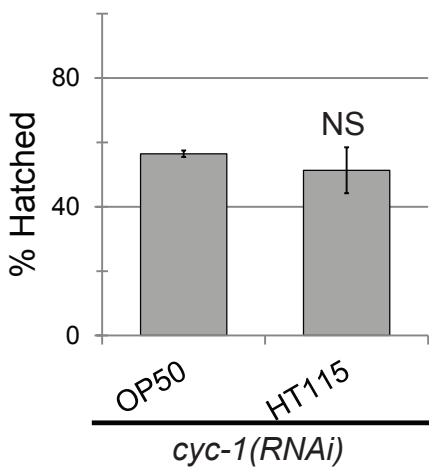

Figure S4

Supplement: S4 Fig — Starting at the L4 stage, C. elegans were fed RNAi-competent strains of E. coli OP50(xu363) or E. coli HT115 bacteria that contained plasmids driving expression of cyc-1 RNAi constructs (which results in embryonic lethality). Each worm was placed onto an individual plate and moved to fresh plate daily. NS–p>0.05 based on Student’s t-test. At least 30 adults were used per biological replicate per condition. Three biological replicates were performed. (PDF) [file pgen.1008011.s004.pdf]

A

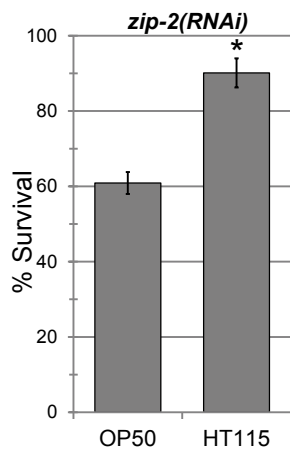

B

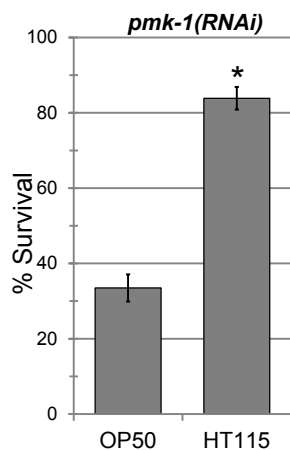

C

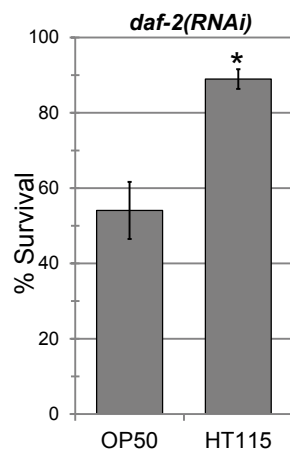

D

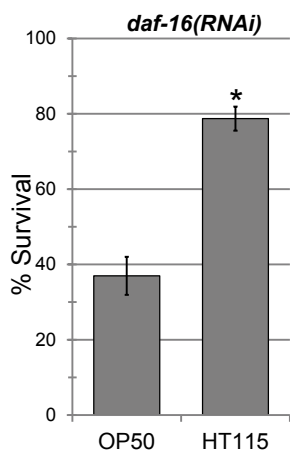

E

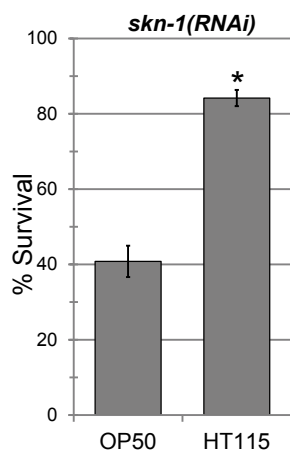

Figure S5

Supplement: S5 Fig — (A-E) Starting at the L1 stage, C. elegans were fed E. coli OP50 or E. coli HT115 bacteria that contained plasmids driving expression of RNAi constructs that targeted innate immune or stress response genes. Young adult worms were subsequently exposed to P. aeruginosa PA14 for an appropriate length of time. Percent survival was inferred based on staining with Sytox Orange, a cell impermeant dye. *—p<0.01 based on Student’s t-test. 10 wells, 20 worms per well, were used per condition per biological replicate. Three biological replicates were performed for each experiment. (PDF) [file pgen.1008011.s005.pdf]

A

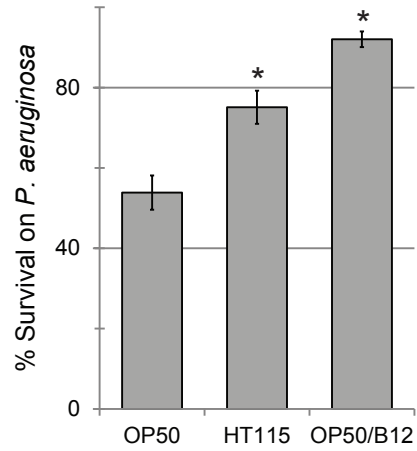

B

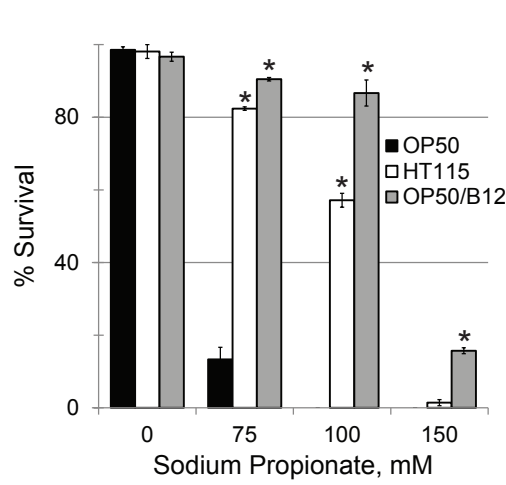

C

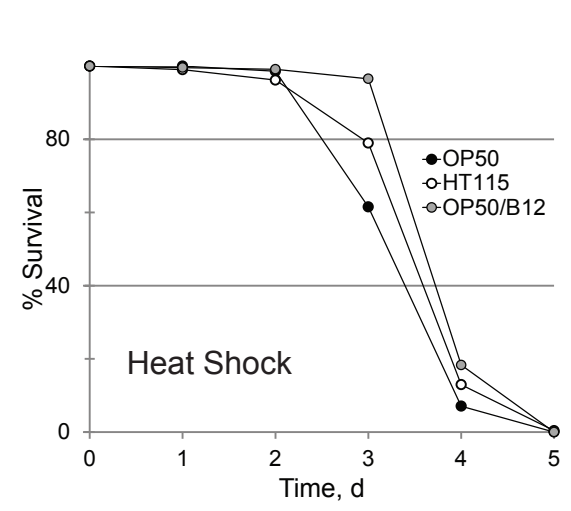

D

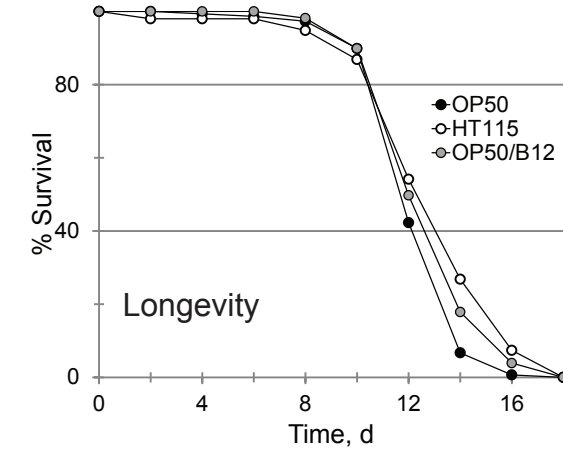

Figure S6

Supplement: S6 Fig — (A-C) Survival of wild-type (N2) C. elegans worms fed E. coli OP50 alone or supplemented with methylcobalamin or E. coli HT115-fed worms in P. aeruginosa-mediated Liquid Killing (A), propionate intoxication (B), or hyperthermia at 30°C (C). (D) Longevity of worms reared as in (A). *—p< 0.01, based on Student’s t-test. For (C, D) p<0.01 was calculated using a log-rank test. For (A), ten wells, with 20 worms per well, were used for each condition for each replicate. For (B-D), 70 (B) or 50 (C, D) worms per plate, three plates per condition were used per replicate. Three biological replicates were performed for each of the experiments. (PDF) [file pgen.1008011.s006.pdf]

A

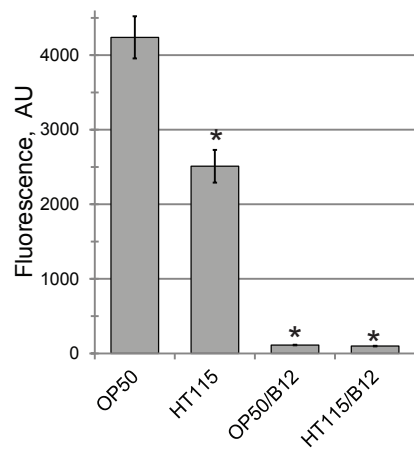

B

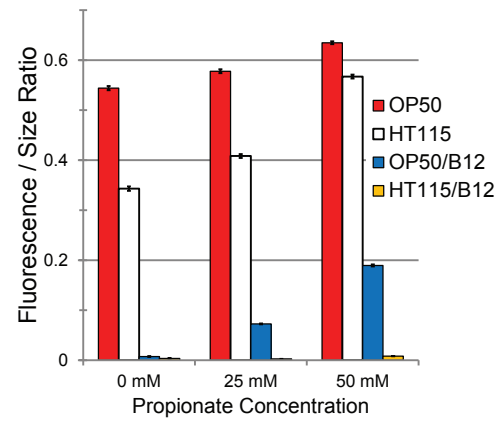

Figure S7

Supplement: S7 Fig — (A) Quantification of acdh-1p::GFP reporter fluorescence in worms fed either E. coli OP50 or E. coli HT115, with or without methylcobalamin supplementation to a final concentration of 200 ng/ml, as indicated. (B) Quantification of acdh-1p::GFP expression in worms fed E. coli OP50 or E. coli HT115, with or without methylcobalamin supplementation to a final concentration of 200 ng/ml and exposed to propionate, using flow vermimetry. Fluorescence for each worm was normalized to its size. *—p<0.01 based on Student’s t-test. For (A) 30 worms per condition per replicate were measured. For (B), 2,000 worms were used per biological replicate per condition, a representative replicate is shown. Three biological replicates were performed for each experiment. (PDF) [file pgen.1008011.s007.pdf]

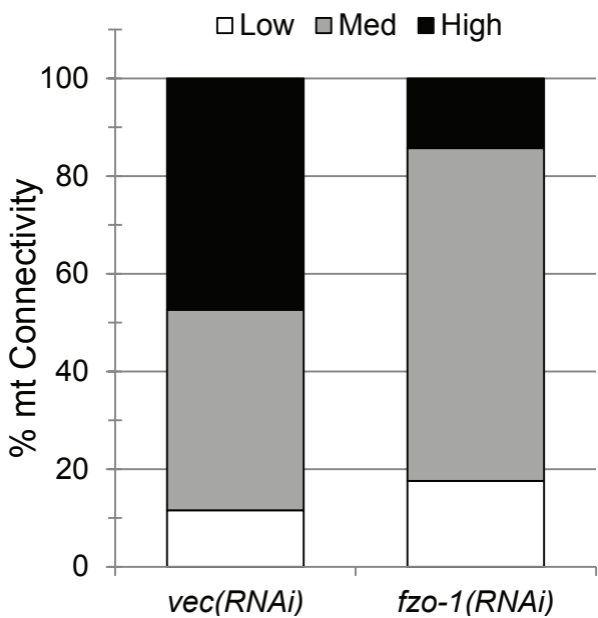

Figure S8

Supplement: S8 Fig — glp-4(bn2ts); myo-3::GFP(mt) worms were reared on either E. coli HT115 containing either an empty RNAi vector or an RNAi vector targeting fzo-1/Mfn1 and then imaged to evaluate fragmentation of the mitochondrial network. Fragmentation was assessed as in Fig 3. 30 worms were used for each condition for each replicate. Three biological replicates were performed for each experiment. (PDF) [file pgen.1008011.s008.pdf]

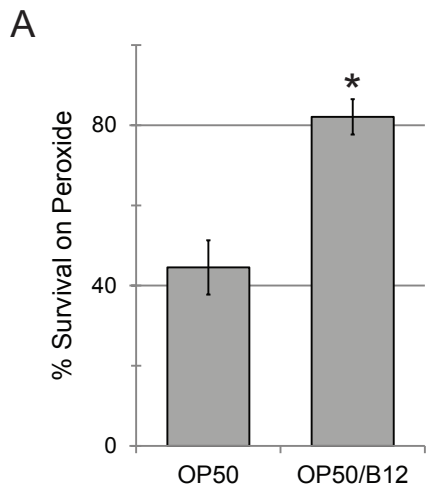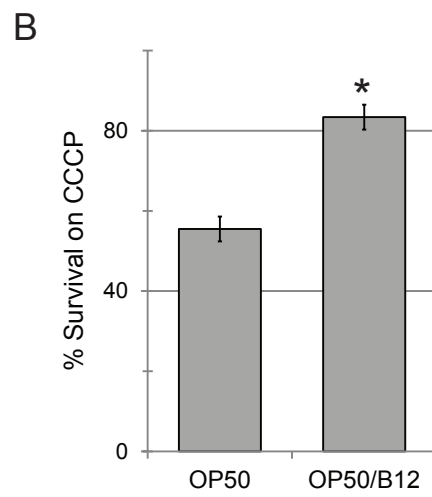

Figure S9

Supplement: S9 Fig — Wild-type (N2) worms were reared on E. coli OP50 with or without methylcobalamin supplementation and then exposed to toxic levels of either peroxide (A) or CCCP (B). 10 wells, with 20 worms per well, were used for each condition for each replicate. At least 2 biological replicates were performed for each experiment. *—p<0.01 based on Student’s t-test. (PDF) [file pgen.1008011.s009.pdf]

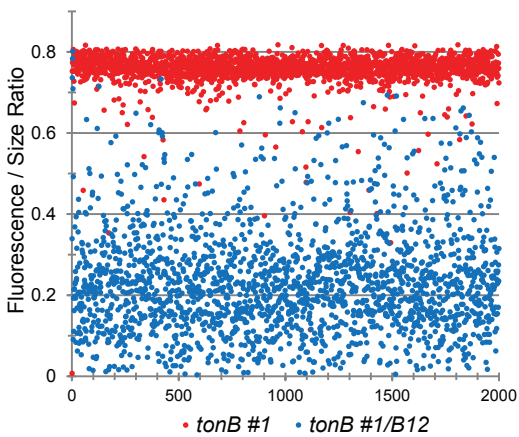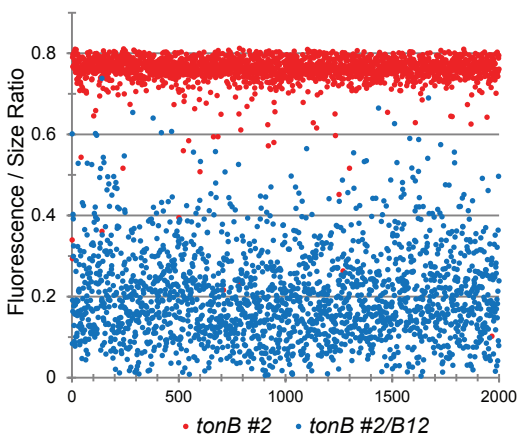

Figure S10

Supplement: S10 Fig — achd-1p::GFP was measured via flow vermimetry in worms reared on two different tonB deletion strains in the E. coli BW25113 background sourced from the Keio collection. Bacteria were grown with and without exogenous methylcobalamin. (PDF) [file pgen.1008011.s010.pdf]

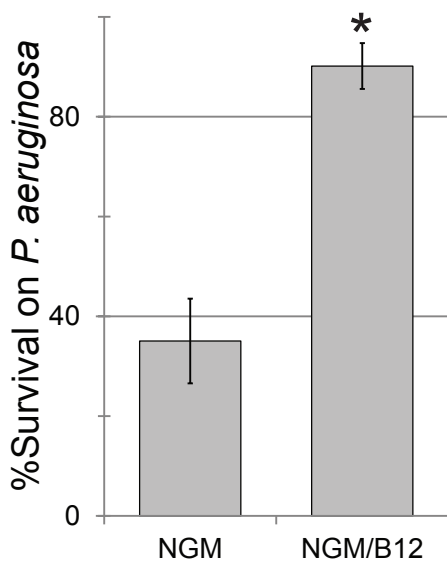

Figure S11

Supplement: S11 Fig — Survival of C. elegans reared on heat-killed E. coli OP50. NGM agar was either supplemented or not with 200 ng/mL of methylcobalamin. Worms were exposed to P. aeruginosa PA14 and survival was assessed after 42 h. (PDF) [file pgen.1008011.s011.pdf]

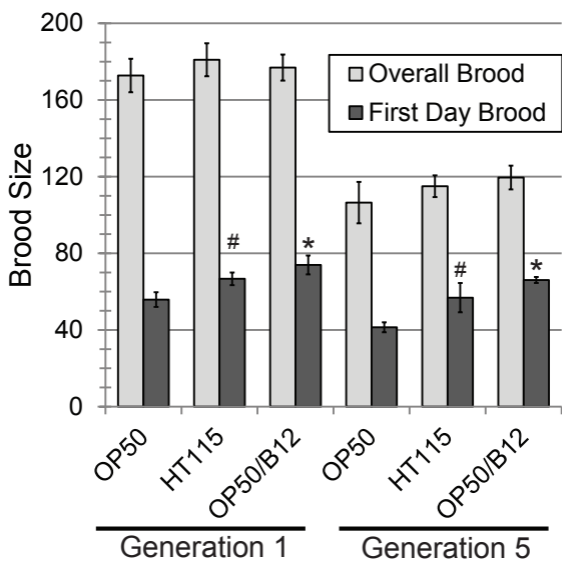

Figure S12

Supplement: S12 Fig — 24h and total brood sizes were determined for wild-type (N2) worms reared on E. coli OP50 with or without exogenous methylcobalamin, or E. coli HT115 that was grown in minimal M9 media. L4 larvae were transferred to fresh plates with the same food, allowed to lay for 24h and then were transferred to a new plate for another 24h. Fecundity was assessed by counting the number of hatched larvae. For each condition, 30 worms were tested per replicate per generation; at least three biological replicates were performed. (PDF) [file pgen.1008011.s012.pdf]
