# Supplementary material for: Interplay between mitochondria and diet mediates pathogen and stress resistance in Caenorhabditis elegans
Source: PLoS Genet. 2019 Mar 13;15(3):e1008011. doi: 10.1371/journal.pgen.1008011 (PMC6415812; doi:10.1371/journal.pgen.1008011)
Supplement: S2 Table — Microarray data on genes that were up- or down-regulated when worms were fed E. coli HT115 (as compared to the standard laboratory diet of E. coli OP50) were compared between [6] and this study. (DOCX) [file pgen.1008011.s014.docx]

**Table S2. Common genes between two transcriptome profiling studies on the effect of HT115 on *C. elegans* transcriptome**

| **ID** | **HT115 Up** | **Gene** | **Name** |
| --- | --- | --- | --- |
| 190619_at | 3.18 | C15C8.3 | *asp-10* |
| 186519_at | 2.24 | F15E11.12 | *pud-4* |
| 187964_at | 4.13 | F54F3.3 | *lipl-1* |
| 183624_at | 2.28 | F55G11.5 | *dod-22* |
| 180256_at | 2.17 | K07E8.3 | *sdz-24* |
| 186182_s_at | 7.65 | R02E12.6 | *hrg-1* |
|  |  |  |  |
| **ID** | **HT115 Down** | **Gene** | **Name** |
| 192528_at | 0.36 | C35A5.3 |  |
| 188822_at | 0.34 | C55B7.4(mt) | *acdh-1* |
| 190067_at | 0.44 | F09F7.4(mt) | *hach-1* |
| 180398_s_at | 0.18 | F15E6.4 |  |
| 192195_at | 0.24 | F28F8.2(mt) | *acs-2* |
| 190145_s_at | 0.49 | F32D8.12(mt) |  |
| 190404_s_at | 0.29 | F37B4.7(mt) | *folt-2* |
| 192692_s_at | 0.28 | F54D5.12(mt) |  |
| 188947_at | 0.38 | T09F5.9 | *clec-47* |
| 193016_s_at | 0.41 | Y38F1A.6(mt) | *hphd-1* |

Note: (mt) marks nuclear genes encoding mitochondrially-localized proteins
